# Non‐Invasive Fibrosis Testing in Adults With Obesity and MASLD: A Systematic Review and Meta‐Analysis

**DOI:** 10.1111/dom.71357

**Published:** 2026-09-17

**Authors:** Patricia M. Ortega, Lucas Sabatella, Pinelopi Manousou, Adriana Arregui, Ahmed R. Ahmed, Chioma Izzi‐Engbeaya

**Affiliations:** ^1^ Department of Surgery Imperial College Healthcare NHS Trust London UK; ^2^ Bariatric Unit, Department of General Surgery Clínica Universidad de Navarra Pamplona Spain; ^3^ Department of Gastroenterology and Hepatology Imperial College Healthcare NHS Trust London UK; ^4^ Department of Metabolism, Digestion and Reproduction Imperial College London London UK; ^5^ Area of Practice Improvement and Innovation in Nursing Clínica Universidad de Navarra Pamplona Spain; ^6^ Department of Surgery and Cancer Imperial College London London UK; ^7^ Department of Endocrinology Imperial College Healthcare NHS Trust London UK

**Keywords:** liver, meta‐analysis, obesity care, systematic review

## Abstract

**Aims:**

Adults with obesity are at increased risk of metabolic dysfunction‐associated steatotic liver disease (MASLD)‐related fibrosis, but the diagnostic performance of non‐invasive tests (NITs) in this population remains uncertain. This meta‐analysis evaluated NITs for detecting significant (≥F2) and advanced (≥F3) fibrosis in adults with obesity and MASLD.

**Materials and Methods:**

PubMed/MEDLINE, Embase, Web of Science and the Cochrane Library were searched from inception to 1 December 2025. Eligible studies enrolled adults with obesity (BMI ≥ 30 kg/m^2^) and MASLD, compared an NIT with liver biopsy, and provided data to estimate sensitivity and specificity. Pooled estimates were derived using bivariate random‐effects models; HSROC curves were generated for each test and fibrosis threshold.

**Results:**

Twenty‐seven studies including 5034 participants were included. Mean age was 40.6 ± 11.4 years and mean BMI was 43.3 ± 7.1 kg/m^2^. For significant fibrosis (≥F2), vibration‐controlled transient elastography (VCTE) showed a sensitivity of 0.64 (95% CI, 0.49–0.77) and specificity of 0.77 (95% CI, 0.66–0.85), with a median threshold of 7.95 kPa (IQR, 7.60–12.00). FIB‐4 showed broadly comparable performance, with a sensitivity of 0.65 (95% CI, 0.53–0.75) and specificity of 0.77 (95% CI, 0.64–0.86). For advanced fibrosis (≥F3), VCTE showed a sensitivity of 0.77 (95% CI, 0.67–0.85) and specificity of 0.84 (95% CI, 0.76–0.90), with a median threshold of 10.73 kPa (IQR, 8.15–12.80). Serum‐based tests showed more variable operating characteristics. Post hoc threshold analyses showed the expected trade‐off between sensitivity and specificity at lower VCTE cut‐offs.

**Conclusions:**

In adults with obesity and MASLD, VCTE showed the most consistent diagnostic performance, particularly for advanced fibrosis, whereas serum‐based tests demonstrated more variable operating characteristics. Threshold selection and technical feasibility remain important determinants of performance, highlighting the need for validated obesity‐specific thresholds and diagnostic pathways.

## 1 Introduction

Metabolic dysfunction–associated steatotic liver disease (MASLD), previously termed non‐alcoholic fatty liver disease (NAFLD) [1], is the most common cause of chronic liver disease worldwide [2] and is a growing global health burden. The rising prevalence of obesity and metabolic syndrome has led to a parallel increase in MASLD including metabolic dysfunction–associated steatohepatitis (MASH), a more active inflammatory phenotype associated with an increased risk of fibrosis progression, cirrhosis and hepatocellular carcinoma, although disease trajectories remain heterogeneous [3]. Steatotic liver disease, including MASLD, has recently been formally recognised within the global non‐communicable disease agenda, reflecting its shared risk factors with obesity, Type 2 diabetes, cardiovascular disease and other metabolic conditions, and highlighting the need for integrated prevention, early detection and management strategies [4]. In populations with obesity, the prevalence of MASLD may exceed 80%, with a substantial proportion of individuals harbouring clinically significant fibrosis despite minimal symptoms [5, 6, 7].

Accurate fibrosis staging is therefore critical for risk stratification and management. Although liver biopsy remains the definitive method for staging and grading MASLD/MASH, its invasive nature, cost and sampling variability limit its applicability for large‐scale screening, particularly in populations with a high prevalence of disease such as individuals with obesity [5, 8, 9].

Consequently, non‐invasive tests (NITs) have been increasingly adopted for fibrosis assessment [10, 11]. However, their diagnostic performance may vary substantially according to the population in which they are applied. Individuals with obesity represent a particularly relevant population because of their high prevalence of MASLD and the metabolic and technical factors that may influence test performance. Previous evidence syntheses have addressed this question but leave important gaps. Ooi et al. [12] evaluated serum biomarkers and elastography techniques in people with obesity and found generally better diagnostic performance with elastography and more complex biomarker panels than with simple serum scores, while also highlighting limited validation of complex panels and BMI‐related technical limitations of transient elastography. More recently, Mózes et al. reported an individual‐participant‐data meta‐analysis showing better discrimination of VCTE than FIB‐4 or NFS for advanced fibrosis [13]. However, that analysis included the broader NAFLD population, with substantially lower BMI than in the present review, and focused on advanced fibrosis and sequential testing strategies. The diagnostic performance of individual NITs across both significant and advanced fibrosis specifically in adults with obesity therefore remains incompletely defined.

Therefore, we conducted a systematic review and diagnostic test accuracy (DTA) meta‐analysis to evaluate individual NITs specifically in adults with obesity. We restricted inclusion to biopsy‐confirmed studies of adults with BMI ≥ 30 kg/m^2^, or studies reporting an extractable subgroup meeting this criterion, and synthesised each index test separately for the detection of significant (≥F2) and advanced (≥F3) fibrosis. This provides an updated, obesity‐specific assessment of commonly used serum‐based and imaging‐based NITs across clinically relevant fibrosis thresholds.

## 2 Methods

This systematic review and diagnostic test accuracy meta‐analysis was conducted following Cochrane recommendations [14] and according to the Preferred Reporting Items for Systematic Reviews and Meta‐Analyses of Diagnostic Test Accuracy Studies (PRISMA‐DTA) guidelines [15]. The protocol was prospectively registered in the PROSPERO database (CRD420261309229).

### 2.1 Search Strategy

A comprehensive literature search was conducted in five databases from inception to December 1, 2025: MEDLINE (via Ovid), Embase (via Ovid), PubMed, Cochrane Library and Web of Science. The strategy included controlled vocabulary and keywords related to obesity, MASLD/NAFLD, liver fibrosis and non‐invasive diagnostic modalities.

No language or geographic restrictions were applied. Reference lists of included studies and relevant reviews were searched manually to identify additional studies. The complete search strategies for each database are available in the Supporting Information.

### 2.2 Study Selection

We included original studies evaluating the diagnostic accuracy of NITs for the detection of histologically confirmed liver fibrosis in adults with obesity and MASLD (previously NAFLD). Eligible studies enrolled participants aged ≥ 18 years in whom obesity, defined as body mass index (BMI) ≥ 30 kg/m^2^, was either an explicit inclusion criterion or for whom a BMI ≥ 30 kg/m^2^ subgroup was separately reported with extractable data.

The reference standard was liver biopsy and assessment of the index test against the reference standard was mandatory for study inclusion. Studies were required to report diagnostic performance for fibrosis‐specific endpoints, where F0 indicates no fibrosis and F4 indicates cirrhosis [15, 16]. Stage ≥F2 is defined as significant fibrosis, ≥F3 as advanced fibrosis and F4 as cirrhosis [16, 17]. Studies evaluating only hepatic steatosis, inflammatory activity without fibrosis‐specific analysis, composite endpoints without separate fibrosis performance or which did not include extractable data were excluded.

All non‐invasive modalities intended to detect liver fibrosis were considered eligible. Composite algorithms were included only if they targeted fibrosis alone, were validated against histology, and provided extractable diagnostic accuracy data.

We excluded screening‐only studies without diagnostic accuracy data, association or correlation studies lacking extractable threshold‐specific diagnostic‐accuracy data, agreement or discordance analyses without test‐level accuracy measures, paediatric cohorts, case reports, conference abstracts and narrative/systematic reviews.

### 2.3 Data Extraction

Two independent reviewers (PO and LS) screened all titles and abstracts using Covidence software (Veritas Health Innovation, Melbourne, Australia), followed by full‐text assessment. Disagreements were resolved through discussion or consultation with a third reviewer (AA, CI).

Extracted variables included study design, country, recruitment setting, sample size, participant demographics (age, sex, BMI), index test characteristics, fibrosis stage distribution and reported diagnostic performance metrics. Raw diagnostic data were extracted to construct 2 × 2 contingency tables (true positives, false positives, false negatives and true negatives) for each index test and fibrosis threshold. When studies did not directly report these values, they were reconstructed from reported sensitivity, specificity, predictive values or receiver operating characteristic data when sufficient information was available.

For feasibility analyses, we additionally extracted, where reported, the numbers of attempted, technically failed, unreliable or invalid and evaluable imaging examinations, together with study‐specific reliability definitions and reported associations with BMI or adiposity. For serum‐based tests, we extracted test availability or calculability and indeterminate classifications when dual‐threshold algorithms were reported. These outcomes were summarised descriptively because definitions and acquisition protocols were heterogeneous.

### 2.4 Quality Assessment

The methodological quality of included studies was assessed independently by two reviewers (PO and AA) using the QUADAS‐2 tool. Risk of bias was evaluated across four domains: patient selection, conduct and interpretation of the index test, conduct and interpretation of the reference standard, and flow and timing. Each domain was rated as low, high or unclear risk of bias. Disagreements were resolved through discussion. Ratings were presented using the *robvis* tool as ‘traffic light’ plots [18].

### 2.5 Statistical Analysis

Diagnostic accuracy was synthesised separately for each index test and fibrosis threshold using a bivariate random‐effects logistic regression model based on study‐level true‐positive, false‐positive, false‐negative and true‐negative counts. Where 2 × 2 counts were not directly reported, they were reconstructed from published diagnostic‐accuracy data only when uniquely recoverable and were cross‐checked against the reported sensitivity and specificity. Models were fitted in Stata using *metandi*. Studies containing zero cells, resulting in observed sensitivity or specificity of 0 or 1, were retained with their original 2 × 2 counts. No continuity correction was applied because the bivariate hierarchical logistic model fitted the binomial data directly rather than relying on logit‐transformed study‐level proportions. Summary sensitivity, specificity, positive and negative likelihood ratios, diagnostic odds ratios (DORs) and hierarchical summary receiver operating characteristic (HSROC) curves were derived from the fitted models.

Between‐study variance–covariance components were estimated by maximum likelihood using adaptive Gaussian quadrature. Approximate Wald‐type 95% confidence intervals were derived from the model‐based variance–covariance matrix on the relevant transformed scale and back‐transformed for presentation; standard errors for transformed HSROC parameters were obtained using the delta method. Numerical stability was assessed by refitting models using 5, 7 and 9 quadrature points and examining the consistency of the resulting estimates. Where the default optimisation procedure failed or produced an unstable Hessian, the equivalent model was refitted using *gllamm* as a numerical sensitivity check. Boundary or near‐boundary estimates of the random‐effects correlation were noted and interpreted cautiously given the small number of contributing studies.

Study influence was assessed using Cook's distance, with 20/k, where *k* was the number of contributing studies, used as a post hoc heuristic screening threshold rather than an exclusion criterion. Standardised empirical Bayes random effects were examined, with absolute values > 2 considered potential residual outliers. Leave‐one‐study‐out analyses were undertaken when omission of one study left at least four studies available for bivariate model estimation. Normal Q‐Q plots of the standardised random effects were examined graphically for gross departures from the assumed distribution. Formal tests of random‐effects normality were not performed because individual models contained only 4–9 studies.

Post hoc sensitivity analyses examined the robustness of pooled estimates to the liver‐biopsy reference standard. The principal restriction excluded studies with known unmitigated wedge or marginal‐wedge sampling and studies in which wedge and core sampling were not separable, while retaining studies with explicit needle/core sampling, explicit mitigation of subcapsular fibrosis, or incompletely reported biopsy technique. A stricter analysis retained studies with explicit needle/core sampling or explicit mitigation of subcapsular fibrosis. Bivariate pooling was not undertaken when fewer than four studies remained. Differences from the primary analyses were interpreted as sensitivity analyses rather than formal subgroup effects. Additional post hoc analyses examined the effect of VCTE threshold selection by restricting analyses to clinically apparent lower threshold clusters; these were interpreted as exploratory sensitivity analyses and not as estimates of optimal obesity‐specific cut‐offs.

All analyses were performed using Stata version 19.5 (StataCorp LLC, College Station, TX, USA).

### 2.6 Role of the Funding Source

This study received no specific funding. The authors were responsible for study design, data collection, data analysis, data interpretation, manuscript preparation and the decision to submit the manuscript for publication.

## 3 Results

The PRISMA flowchart is shown in Figure S1. A total of 14 202 records were identified through database searching. After removal of 5037 duplicates, 9165 records remained for title and abstract screening. Of these, 778 reports were retrieved for full‐text assessment. Following full‐text review, 751 studies were excluded. Ultimately, 27 studies met the inclusion criteria and were included in the final analysis.

### 3.1 Study Characteristics

The characteristics of the included studies are detailed in Table 1. A total of 27 studies [19, 20, 21, 22, 23, 24, 25, 26, 27, 28, 29, 30, 31, 32, 33, 34, 35, 36, 37, 38, 39, 40, 41, 42, 43, 44, 45] comprising 5034 participants were included in the analysis. Most studies were prospective observational studies conducted in bariatric surgery cohorts.

**TABLE 1:** Study characteristics.

Study	Country	Study design	Setting	*n* (Total)	Mean age ± SD (years)	% Female	Mean BMI ± SD (kg/m^2^)	Index test	≥F2 (%)	≥F3 (%)
Agarwal 2021	USA	Prospective	Bariatric	179	39.2 ± NR	78	45.2 ± 7.1	Fibroscan (VCTE)	36	17
Alcantara‐Diaz 2023	Peru	Retrospective	Bariatric	227	37.8 ± 11.1	65	36.8 ± 5.5	2D‐SWE	44	8
Ali 2022	USA	Prospective	Bariatric	167	46.4 ± 13.6	83	48 ± 6.8	Fibroscan (VCTE)	11	7
Barsamian 2020	France	Prospective	Bariatric	116	41 ± 12.6	79	43 ± 9	Fibroscan (VCTE)	19	10
Bauer 2024	Austria	Prospective	Bariatric, Hepatology	129	44 ± 17	64	43.2 ± 11.4	VCTE, pSWE, 2D‐SWE	47	8
Chen 2017	USA	Prospective	Bariatric, Hepatology	111	48.1 ± 11.7	66	40.3 ± 1.5	MRE, VCTE	20	8
DaSilva 2021	Brazil	Prospective	Bariatric	108	44.6 ± 10.4	79	46.9 ± 6.6	ARFI, APRI, FIB‐4, Forn, NFS, BARD	19	8
DeCarli 2020	Brazil	Retrospective	Bariatric	323	36.9 ± 9.1	82	44.2 ± 5.6	AAR, APRI, BARD, FIB‐4, NFS, BAAT	NR	9
Dominik 2025	Austria	Prospective	Bariatric, Hepatology	134	42.6 ± 13.3	65	42.7 ± 10.4	Auto pSWE, VCTE, UDFF, CAP	28	7
Eilenberg 2021	Austria	Retrospective	Bariatric	170	42 ± 12.7	65	44.4 ± 5.6	VCTE, CAP, FAST score	22	8
Garg 2018	India	Prospective	Bariatric	76	39.3 ± 10.9	77	45.2 ± 7.1	Fibroscan (VCTE)	39	14
Giraudi 2018	Italy	Prospective	Bariatric	71	43 ± 12	66	44 ± 7	IGF2, EGFR, EGFR/IGF2 ratio, FIB‐4, FibroMeter	18	1
Giraudi 2022	Italy	Retrospective	Bariatric	235	45 ± 10	67	43.9 ± 5.8	FCN‐2, FCN‐2 + APRI + HDL	24	5
Huang 2023	China	Prospective	Bariatric	373	30.9 ± 9	66	39.4 ± 7.6	APRI, FIB‐4, NFS, BARD, HFS	9	4
Jamialahmadi 2019	Iran	Prospective	Bariatric	90	38.5 ± 11.1	80	45.5 ± 6.3	2D‐SWE	13	6
Lassailly 2011	France	Prospective	Bariatric	288	41.6 ± 12.8	77	48.6 ± 8.9	FibroTest, SteatoTest, ActiTest, NashTest	7	2
Lefere 2017	Belgium	Prospective	Bariatric	61	43 ± 14.1	0	41.3 ± 4.8	Serum VCAM‐1	33	NR
Marques 2021	Portugal, Spain	Prospective	Bariatric	194	47 ± 14.5	66	44.6 ± 0.7	Leptin, adiponectin, IGF‐1 panels	NR	10
Nassif 2017	Brazil	Retrospective	Bariatric	298	40.1 ± NR	89	43.6 ± NR	BARD	7	NR
Naveau 2014	France	Prospective	Bariatric	100	42.5 ± 5	81	42.3 ± 0.5	Fibroscan (VCTE)	22	9
Ooi 2017	Australia	Prospective	Bariatric	101	49 ± 11.9	66	41.9 ± 5.8	APRI, Forn, FIB‐4, NFS, BARD	23	3
Pimentel 2010	Brazil	Prospective	Bariatric	158	36 ± 10	77	41 ± 5	NFS	62	14
Schmitz 2020	Germany	Prospective	Bariatric	141	44 ± 9	73	53 ± 7	APRI, FIB‐4, NFS, NCS, LiMAx	29	8
Smiderle 2021	Brazil	Retrospective	Bariatric	423	37.1 ± 9.7	79	43.8 ± 5.6	TyG Index, NFS	12	8
Soresi 2020	Italy	Retrospective	Bariatric	57	42 ± 12	72	43.5 ± 7.1	BARD, NFS, FIB‐4, US	37	5
Sun 2025	China	Retrospective	Bariatric	241	30.8 ± 7.8	70.1	40.8 ± 6.4	Auto pSWE, FIB‐4, APRI	14	2
Younossi 2023	USA	Retrospective	Hepatology	463	48 ± 13	69	40.7 ± 10	ELF, FIB‐4	39	NR

*Note:* Standard deviation (SD) values were estimated from ranges, interquartile ranges or standard errors when not reported. ≥F2 (%) indicates the proportion of patients with significant fibrosis and ≥F3 (%) indicates the proportion with advanced fibrosis, based on histological assessment on liver biopsy.

Abbreviations: 2D‐SWE, two‐dimensional shear wave elastography; AAR, AST‐to‐ALT ratio; APRI, AST‐to‐platelet ratio index; ARFI, acoustic radiation force impulse elastography; BAAT, BMI‐age‐ALT‐triglycerides score; BARD, BMI‐AST/ALT ratio‐diabetes score; CAP, controlled attenuation parameter; EGFR, epidermal growth factor receptor; ELF, enhanced liver fibrosis score; FAST, FibroScan‐AST score; FCN‐2, ficolin‐2; FIB‐4, fibrosis‐4 index; HFS, Hepamet fibrosis score; IGF‐1, insulin‐like growth factor‐1; IGF‐2, insulin‐like growth factor‐2; LiMAx, liver maximum capacity test; MRE, magnetic resonance elastography; NCS, Non‐alcoholic steatohepatitis (NASH) clinical score; NFS, non‐alcoholic fatty liver disease (NAFLD) fibrosis score; NR, not reported; pSWE, point shear wave elastography; TyG, triglyceride‐glucose index; US, ultrasound; UDFF, ultrasound‐derived fat fraction; VCAM‐1, vascular cell adhesion molecule‐1; VCTE, vibration‐controlled transient elastography.

The pooled mean age was 40.6 ± 11.4 years, the pooled mean BMI was 43.3 ± 7.1 kg/m^2^, and study‐level mean BMI ranged from 36.8 to 53.0 kg/m^2^. Overall, 72.6% of participants were female.

Imaging‐based modalities included vibration‐controlled transient elastography (VCTE), magnetic resonance elastography (MRE), two‐dimensional shear wave elastography (2D‐SWE), point shear wave elastography (pSWE) and automated point shear wave elastography (Auto‐pSWE). Serum‐based biomarkers included commonly used fibrosis risk scores such as fibrosis‐4 index (FIB‐4), non‐alcoholic fatty liver disease fibrosis score (NFS), AST‐to‐platelet ratio index (APRI) and the BMI‐AST/ALT ratio‐diabetes (BARD) score. Study‐level diagnostic accuracy data, including reconstructed 2 × 2 contingency tables and derived measures of sensitivity, specificity, and likelihood ratios, are provided in Table S1 and Table S2.

### 3.2 Diagnostic Accuracy for Detection of Significant Fibrosis (≥F2) in People With Obesity

Four NITs (VCTE, FIB‐4, NFS and APRI) were evaluated for the detection of significant fibrosis (≥F2). Summary diagnostic accuracy estimates are presented in Table 2.

**TABLE 2:** Pooled diagnostic accuracy for detection of significant fibrosis (≥F2).

Test	Studies (*n*)	Participants^a^ (*n*)	Sensitivity^b^ (95% CI)	Specificity^b^ (95% CI)	DOR (95% CI)
VCTE	9	1059	0.64 (0.49–0.77)	0.77 (0.66–0.85)	5.92 (4.17–8.40)
FIB‐4	7	1375	0.65 (0.53–0.75)	0.77 (0.64–0.86)	5.98 (3.92–9.14)
NFS	4	639	0.56 (0.37–0.73)	0.74 (0.50–0.89)	3.53 (1.96–6.34)
APRI	4	823	0.72 (0.61–0.81)	0.63 (0.48–0.76)	4.41 (2.52–7.71)

Abbreviations: APRI, aspartate aminotransferase‐to‐platelet ratio index; DOR, diagnostic odds ratio; FIB‐4, Fibrosis‐4 index; NFS, NAFLD fibrosis score; VCTE, vibration‐controlled transient elastography.

^a^ Participants represent the sum of test‐specific evaluable participants contributing to each pooled analysis.

^b^ Sensitivity and specificity estimates were pooled using a bivariate random‐effects model.

VCTE was evaluated across nine studies [19, 21, 22, 23, 24, 27, 28, 29, 38] and 1059 patients. The pooled sensitivity was 0.64 (95% CI 0.49–0.77) and specificity was 0.77 (95% CI 0.66–0.85), corresponding to a DOR of 5.92 (95% CI 4.17–8.40). VCTE thresholds ranged from 6.30 to 13.30 kPa, with a median of 7.95 kPa (IQR 7.60–12.00).

Among serum‐based biomarkers, FIB‐4 was evaluated in seven studies [31, 33, 40, 42, 44, 45, 46] with a sensitivity of 0.65 (95% CI 0.53–0.75), specificity of 0.77 (95% CI 0.64–0.86), and DOR of 5.98 (95% CI 3.92–9.14). NFS, assessed in four studies [25, 32, 39, 43] had lower sensitivity (0.56; 95% CI 0.37–0.73) and a specificity of 0.74 (95% CI 0.50–0.89), with a DOR of 3.53 (95% CI 1.96–6.34). APRI, evaluated in four studies [25, 32, 39, 44], demonstrated higher sensitivity (0.72; 95% CI 0.61–0.81) but lower specificity (0.63; 95% CI 0.48–0.76), yielding a DOR of 4.41 (95% CI 2.52–7.71).

The HSROC curves for the detection of significant fibrosis are presented in Figure 1. Visual inspection demonstrated relatively concentrated study‐level estimates around the summary operating points for VCTE and FIB‐4, whereas NFS and APRI showed greater dispersion across the sensitivity–specificity space and visibly broader confidence and prediction regions.

**FIGURE 1:**
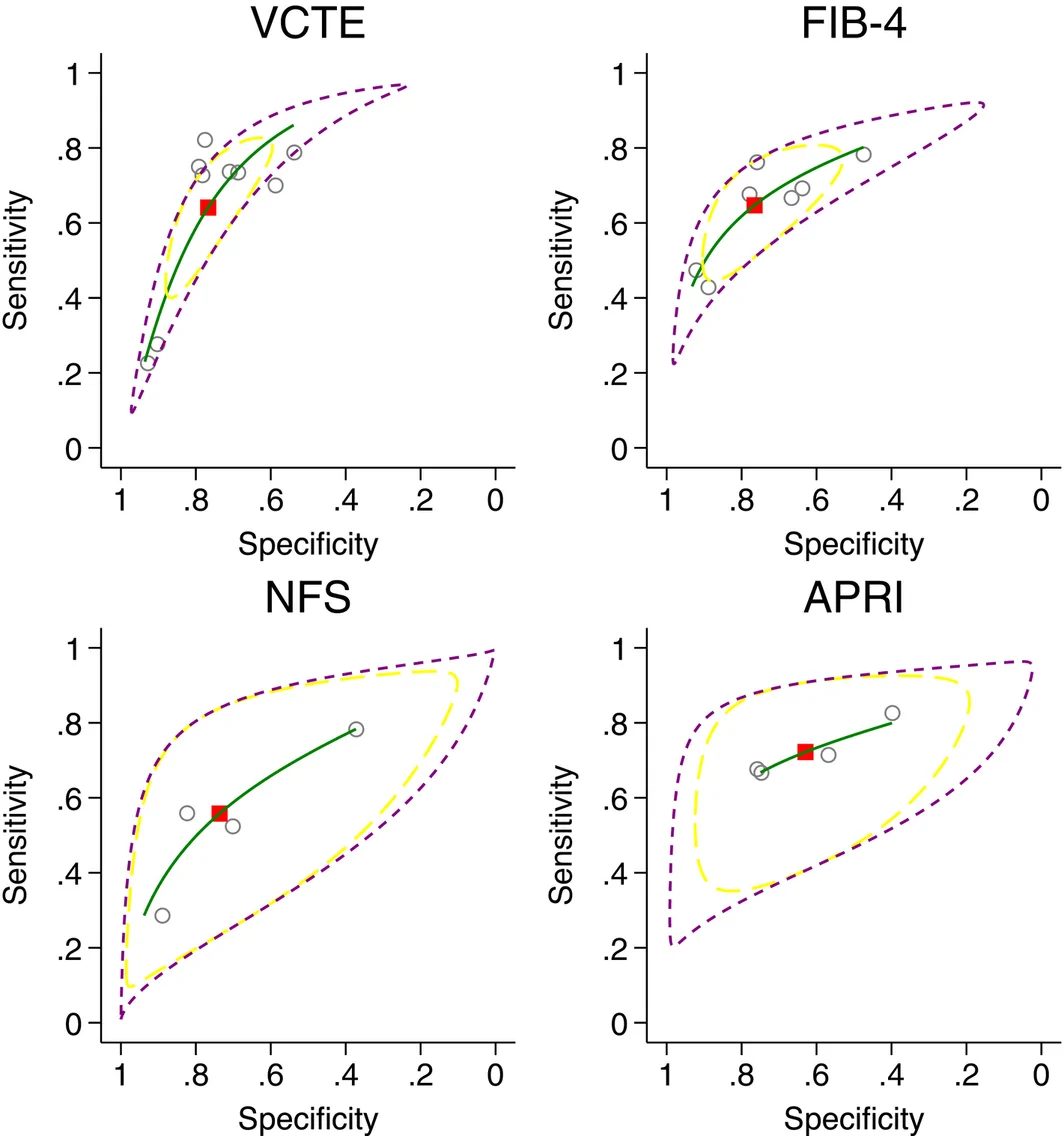
HSROC curves for non‐invasive tests detecting significant fibrosis (≥F2). Each panel displays study‐specific estimates of sensitivity and specificity (open circles), the pooled summary operating point (red square), and the HSROC curve (green line). The dashed yellow ellipse represents the 95% confidence region around the pooled estimate, while the dashed purple ellipse represents the 95% prediction region. The *x*‐axis represents specificity (reversed scale) and the *y*‐axis represents sensitivity.

### 3.3 Diagnostic Accuracy for Detection of Advanced Fibrosis (≥F3) in People With Obesity

Four NITs were evaluated for detecting advanced fibrosis: VCTE, FIB‐4, NFS and APRI. Summary diagnostic accuracy estimates are presented in Table 3.

**TABLE 3:** Pooled diagnostic accuracy for detection of advanced fibrosis (≥F3).

Test	Studies (*n*)	Participants^a^ (*n*)	Sensitivity^b^ (95% CI)	Specificity^b^ (95% CI)	DOR (95% CI)
VCTE	8	920	0.77 (0.67–0.85)	0.84 (0.76–0.90)	18.12 (10.52–31.22)
FIB‐4	5	856	0.63 (0.40–0.82)	0.76 (0.52–0.90)	5.54 (2.53–12.11)
NFS	6	1061	0.47 (0.21–0.75)	0.89 (0.76–0.95)	7.14 (1.47–34.79)
APRI	5	871	0.59 (0.36–0.78)	0.92 (0.74–0.98)	15.40 (5.84–40.60)

Abbreviations: APRI, aspartate aminotransferase‐to‐platelet ratio index; DOR, diagnostic odds ratio; FIB‐4, Fibrosis‐4 index; NFS, NAFLD fibrosis score; VCTE, vibration‐controlled transient elastography.

^a^ Participants represent the sum of test‐specific evaluable participants contributing to each pooled analysis.

^b^ Sensitivity and specificity estimates were pooled using a bivariate random‐effects model.

VCTE was evaluated across eight studies [19, 22, 23, 24, 27, 28, 29, 38] and 920 test‐evaluable participants, with a pooled sensitivity of 0.77 (95% CI 0.67–0.85) and specificity of 0.84 (95% CI 0.76–0.90), corresponding to a DOR of 18.12 (95% CI 10.52–31.21). Study‐specific VCTE thresholds ranged from 7.60 to 15.00 kPa, with a median of 10.73 kPa (IQR 8.15–12.80).

Among serum‐based tests, FIB‐4 was evaluated in five studies [26, 36, 39, 41, 44] comprising 856 participants, with a pooled sensitivity of 0.63 (95% CI 0.40–0.82), specificity of 0.76 (95% CI 0.52–0.90) and DOR of 5.54 (95% CI 2.53–12.11). NFS, evaluated in six studies [26, 36, 39, 40, 41, 42] comprising 1061 participants, showed lower sensitivity (0.47; 95% CI 0.21–0.75) but higher specificity (0.89; 95% CI 0.76–0.95), yielding a DOR of 7.14 (95% CI 1.47–34.79). APRI, evaluated in five studies [26, 36, 39, 41, 44] comprising 871 participants, showed sensitivity of 0.59 (95% CI 0.36–0.78) and the highest pooled specificity (0.92; 95% CI 0.74–0.98), with a DOR of 15.40 (95% CI 5.84–40.60).

The HSROC curves for advanced fibrosis are shown in Figure 2. Visual inspection showed relatively concentrated study‐level estimates around the VCTE summary operating point, whereas FIB‐4, NFS and APRI exhibited greater dispersion across the sensitivity–specificity space. The NFS model showed particularly wide confidence and prediction regions, consistent with substantial uncertainty in the pooled estimate, while APRI combined relatively high specificity with greater between‐study dispersion.

**FIGURE 2:**
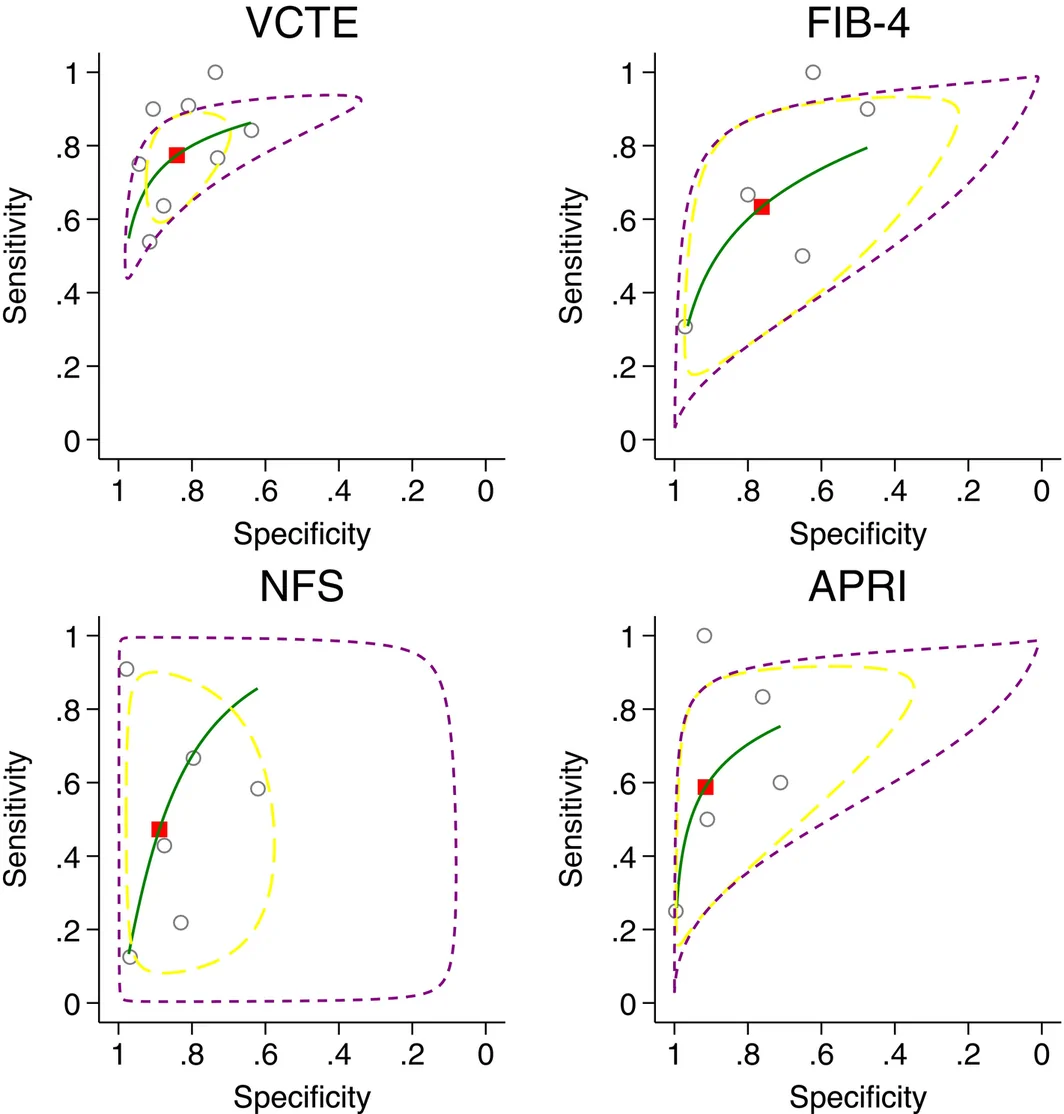
HSROC curves for non‐invasive tests detecting advanced fibrosis (≥F3). Each panel displays study‐specific estimates of sensitivity and specificity (open circles), the pooled summary operating point (red square), and the HSROC curve (green line). The dashed yellow ellipse represents the 95% confidence region around the pooled estimate, while the dashed purple ellipse represents the 95% prediction region. The *x*‐axis represents specificity (reversed scale) and the *y*‐axis represents sensitivity.

Several NITs were evaluated in individual studies but were not included in pooled analyses due to the limited number of available studies. These included imaging‐based techniques such as MRE, ultrasound‐derived elastography methods, several serum‐based biomarkers (e.g., the enhanced liver fibrosis (ELF), BARD score) and exploratory panels (IGF‐related markers, FCN‐2, VCAM‐1, TyG index and combination biomarker panels).

### 3.4 Model Diagnostics and Post Hoc Sensitivity Analysis

Model diagnostics generally supported the numerical stability and robustness of the pooled estimates. No study exceeded the standardised empirical Bayes screening threshold; no study exceeded the Cook's distance heuristic for ≥F2, and only Pimentel et al. did so in the ≥F3 NFS model. Leave‐one‐study‐out analyses, where estimable, did not change the overall clinical interpretation, and Q–Q plots showed no gross departure from the assumed random‐effects distribution.

Post hoc biopsy‐reference‐standard restrictions produced only modest changes in VCTE sensitivity, while specificity and DORs remained broadly stable; restricted ≥F3 NFS estimates were imprecise. Some small‐study models yielded boundary random‐effects correlation estimates and were therefore interpreted cautiously.

Study‐specific VCTE thresholds varied substantially. Post hoc analyses restricted to the lower threshold clusters at both fibrosis endpoints showed higher sensitivity and lower specificity than the corresponding primary analyses, consistent with the expected threshold‐related trade‐off, while overall discrimination was broadly preserved. Threshold‐stratified analyses were exploratory, and the higher threshold ≥F3 subset could not be stably estimated because of the small number of studies.

Detailed results are provided in Tables S3, S4, S6 and S7.

### 3.5 Practical Feasibility and Indeterminate Classifications

Practical feasibility varied substantially across imaging modalities and study‐specific definitions of technical failure and reliability. For elastography‐based tests, unsuccessful or unreliable examinations were most consistently associated with greater adiposity, including higher BMI, abdominal or waist circumference and subcutaneous tissue thickness; newer deep‐abdominal and automated shear‐wave techniques reduced acquisition failure in some studies, although unreliable measurements could still occur. For serum‐based tests, applicability was primarily limited by missing components required for score calculation and by indeterminate or unresolved classifications when dual‐threshold strategies were used; the extent of this problem varied considerably between tests and studies, and in some reports could not be estimated from the published data. Detailed study‐level feasibility, availability and indeterminate‐classification data are presented in Table S5.

### 3.6 Quality Assessment

Most studies were judged at low risk of bias for patient selection and reference standard, reflecting recruitment from bariatric cohorts without case–control designs and the consistent use of liver biopsy with validated histological scoring systems. The index test domain was frequently rated as high risk of bias, mainly because thresholds were often derived from ROC analyses within the same study rather than pre‐specified. The flow and timing domain was generally low or unclear risk, as NITs were usually performed shortly before intraoperative liver biopsy, although incomplete reporting of exclusions, missing data or test failures introduced some uncertainty. The overall QUADAS‐2 assessment is shown in the traffic light plot (Figure S2).

## 4 Discussion

Three key findings emerge from this analysis. First, VCTE showed the most consistent diagnostic performance, particularly for advanced fibrosis, whereas its performance for significant fibrosis was broadly comparable with FIB‐4. Second, serum‐based tests showed more variable operating characteristics, with differences largely reflecting trade‐offs between sensitivity and specificity rather than clear superiority of one marker across endpoints. Third, VCTE performance was influenced by threshold selection: lower cut‐offs increased sensitivity at the expense of specificity, while overall discrimination was broadly preserved. These findings highlight the importance of threshold choice in this population and underscore the need for externally validated, obesity‐specific VCTE cut‐offs.

Current clinical guidelines advocate a stepwise strategy for fibrosis risk assessment in individuals with MASLD [10, 11], typically using simple serum‐based scores such as FIB‐4 for initial risk stratification, followed by elastography in patient with indeterminate or elevated results. This strategy reflects the complementary roles of these tests: serum‐based scores are inexpensive and widely accessible, whereas elastography provides more direct assessment of liver stiffness and showed particularly strong discrimination for advanced fibrosis in our analysis. The recently updated American Gastroenterological Association MASLD Clinical Care Pathway retains FIB‐4 as a first‐tier test while recognising that direct progression to imaging‐based NITs may be reasonable when these are readily available, particularly in higher‐risk settings [46]. Obesity may affect both components of this pathway: metabolic and biochemical alterations may influence serum‐based scores [47, 48], while increased skin‐to‐liver distance and subcutaneous adiposity can impair ultrasound‐based elastography [49, 50]. Our feasibility findings confirm that technical failure and unreliable elastography measurements remain relevant in this population despite improvements in probe and acquisition technology. Taken together, our results support a complementary rather than competing role for serum‐based and imaging‐based NITs in adults with obesity, with test selection guided by clinical risk, local availability and technical feasibility.

Several studies have reported that the performance of commonly used serum‐based fibrosis scores may be diminished in individuals with obesity, particularly in those with severe obesity [12, 13, 51, 52, 53], due to alterations in transaminase levels, platelet counts and systemic inflammatory markers [47, 48]. Elastography‐based techniques such as VCTE have generally shown stronger discrimination for advanced fibrosis [54, 55, 56, 57]. With a pooled mean BMI exceeding 40 kg/m^2^ and a large proportion of bariatric cohorts, our analysis extends previous evidence by evaluating commonly used NITs specifically in a population with severe obesity that remains under‐represented in broader MASLD diagnostic‐accuracy studies [12, 13].

Importantly, our findings suggest that diagnostic discrimination may improve as fibrosis becomes more advanced, a pattern that was most evident for VCTE. Similar observations have been reported in broader MASLD populations and may reflect the stronger biological and structural signals associated with more advanced fibrotis [13, 58, 59, 60]. However, evidence derived specifically from populations with obesity remains limited [12, 60, 61, 62], making the present obesity‐focused analysis particularly relevant. Accurate detection of earlier‐stage fibrosis remains more challenging but clinically important, as it may allow intervention before progression to advanced disease [63]. Future studies should therefore prioritise the validation of NITs and clinically useful thresholds for detecting significant fibrosis in individuals with obesity.

Several limitations should be acknowledged. First, the included studies were heterogeneous in study design, patient characteristics and diagnostic thresholds, which may have influenced the pooled estimates. Model diagnostics were also constrained by the small number of studies contributing to each analysis. Several models produced boundary estimates of the sensitivity‐specificity random‐effects correlation, reflecting limited identification of the variance–covariance structure, although pooled estimates were generally numerically stable across alternative model specifications. Influence and residual diagnostics did not identify systematic evidence that individual studies drove the findings, although Pimentel et al. [40] was influential in the ≥F3 NFS model. Finally, the threshold‐restricted VCTE analysis was post hoc and based on small study subsets; in particular, the higher threshold ≥F3 model could not be estimated. These analyses should therefore be considered supportive and exploratory rather than definitive evidence for specific diagnostic thresholds.

BMI was generally reported only as an aggregate study‐level measure, precluding assessment of diagnostic performance based on obesity classes or derivation of BMI‐specific thresholds. Although differences in study‐level BMI and clinical settings may have contributed to heterogeneity, meta‐regression was not considered sufficiently robust given the small number of studies available for most index tests and the marked imbalance between bariatric and non‐bariatric cohorts. Similarly, potential differences according to race or ethnicity could not be examined because these characteristics were inconsistently reported. Individual‐participant data or prospectively stratified studies will be required to address these questions. Differences in elastography techniques, diagnostic thresholds and fibrosis staging may have contributed to between‐study variability, particularly as several studies derived thresholds within the same cohorts rather than applying prespecified cut‐offs, introducing potential optimism bias. Most studies were conducted in metabolic and bariatric surgery populations, which may limit generalisability to other clinical settings. Liver biopsy was also an imperfect reference standard because of sampling and interpretative variability: some bariatric cohorts used wedge or subcapsular sampling, although post hoc restrictions by biopsy technique produced only modest changes in VCTE estimates and did not suggest major distortion of the pooled findings. Finally, several biomarkers and non‐VCTE imaging modalities were evaluated in too few studies for quantitative synthesis.

Diagnostic accuracy among evaluable participants may not fully reflect real‐world test yield. Imaging‐based NITs remain susceptible to technical failure or unreliable measurements, particularly with greater adiposity, whereas serum‐based scores may be limited by missing components and indeterminate classifications. These practical limitations should be considered alongside diagnostic accuracy when designing clinical pathways. Future prospective studies should therefore report intention‐to‐diagnose performance, technical feasibility and indeterminate classifications in addition to conventional sensitivity and specificity.

Evidence for less commonly studied NITs remains limited. In addition, reliance on aggregate study‐level data precluded assessment of whether diagnostic performance varied according to clinically relevant characteristics such as age, Type 2 diabetes or obesity class. Recent evidence suggests that the performance of some NITs may differ across demographic and metabolic subgroups [57], highlighting the need for individual‐participant data analyses to refine subgroup‐specific thresholds and diagnostic pathways in obesity.

In conclusion, VCTE showed the most consistent diagnostic performance in adults with obesity, particularly for advanced fibrosis, whereas serum‐based scores demonstrated more variable operating characteristics. Threshold selection, technical feasibility and indeterminate classifications remain important determinants of real‐world performance, supporting a complementary rather than single‐test approach to fibrosis assessment. Further prospective studies should validate obesity‐specific thresholds and diagnostic pathways, particularly for the detection of significant fibrosis.

## Author Contributions

Conceptualisation: P.M.O., L.S., A.A. Data acquisition: P.M.O., L.S., A.A., C.I.‐E. Data Analysis: P.M.O. Data Interpretation: All authors. Writing – original draft: P.M.O. Writing – review and editing: All authors.

## Funding

This work was supported by NIHR Imperial Biomedical Research Centre, Medical Research Council, National Institute for Health and Care Research and Efficacy and Mechanism Evaluation Programme.

## Conflicts of Interest

Chioma Izzi‐Engbeaya has received consulting fees from Novo Nordisk and Boehringer Ingelheim unrelated to the submitted work. All other authors declare no conflicts of interest.

## Supporting information


**Figure S1:** PRISMA flowchart.
**Figure S2:** Quality assessment. Risk of bias assessment of included studies using the QUADAS‐2 tool. Traffic‐light plot summarising the methodological quality of the included studies across the four QUADAS‐2 domains: patient selection (D1), index test (D2), reference standard (D3), and flow and timing (D4). Each circle represents the judgement for an individual study within a given domain. Green circles indicate low risk of bias, yellow circles indicate uncertainty, and red circles indicate high risk of bias. The overall risk of bias judgement for each study is shown in the final column.
**Table S1:** Study‐level diagnostic accuracy data for detection of significant fibrosis (≥F2).
**Table S2:** Study‐level diagnostic accuracy data for detection of advanced fibrosis (≥F3).
**Table S3:** Model diagnostics and influence analyses for detection of significant fibrosis (≥F2).
**Table S4:** Model diagnostics and influence analyses for detection of advanced fibrosis (≥F3).
**Table S5:** Technical feasibility, non‐evaluable examinations and indeterminate classifications of NITs.
**Table S6:** Liver biopsy details.
**Table S7:** Post hoc VCTE threshold sensitivity analyses.

## Data Availability

The data that support the findings of this study are available from the corresponding author upon reasonable request.
