# Supplementary material for: Non‐Invasive Fibrosis Testing in Adults With Obesity and MASLD: A Systematic Review and Meta‐Analysis
Source: Diabetes Obes Metab. 2026 Sep 17;28(12):11578–90. doi: 10.1111/dom.71357 (PMC13731011; doi:10.1111/dom.71357)
Supplement: Supplementary file 1 [file DOM-28-11578-s001.docx]

Supplementary material

[1.	SEARCH STRATEGY	4](#_Toc238455647)

[2.	SUPPLEMENTARY DATA	10](#_Toc238455648)

[**eFigure 1. PRISMA Flowchart**	11](#_Toc238455649)

[**eFigure 2. Quality assessment**	12](#_Toc238455650)

[**eTable 1. Study-level diagnostic accuracy data for detection of significant fibrosis (≥F2)**	14](#_Toc238455652)

[**eTable 2. Study-level diagnostic accuracy data for detection of advanced fibrosis (≥F3)**	16](#_Toc238455653)

[**Model diagnostics and influence analysis**	18](#_Toc238455654)

[**eTable 3. Model diagnostics and influence analyses for detection of significant fibrosis (≥F2)**	18](#_Toc238455655)

[**eTable 4. Model diagnostics and influence analyses for detection of advanced fibrosis (≥F3)**	20](#_Toc238455656)

[**eTable 5. Technical feasibility, non-evaluable examinations and indeterminate classifications of NITs**	21](#_Toc238455657)

[**eTable 6. Liver biopsy details**	25](#_Toc238455658)

[**eTable 7. Post-hoc VCTE threshold sensitivity analyses**	31](#_Toc238455659)

## 1. SEARCH STRATEGY

**MEDLINE Via Ovid**

1	exp Non-alcoholic Fatty Liver Disease/	29842

2	(NAFLD or NASH or MASLD or MAFLD or "metabolic dysfunction-associated steatotic liver disease").ti,ab.	43876

3	"Non-alcoholic Steatohepatitis".mp.	7393

4	1 or 2 or 3	51160

5	exp Liver Cirrhosis/	110161

6	exp Liver Fibrosis/	110161

7	(fibrosis or fibrotic or "liver fibrosis" or "hepatic fibrosis" or cirrhosis or "liver cirrhosis" or F2 or F3 or F4).ti,ab.	426267

8	5 or 6 or 7	455340

9	exp Obesity/	289050

10	exp Body Mass Index/	163785

11	(obes* or BMI or "body mass index" or "morbid obes*" or "severe obes*" or "class I obes*" or "class II obes*" or "class III obes*" or bariatric or "weight loss surgery" or "bariatric surgery").ti,ab.	699484

12	9 or 10 or 11	769595

13	exp Biomarkers/	970444

14	exp procedures/	751528

15	exp Sensitivity/ and Specificity/	383141

16	(noninvasive or "non-invasive" or biomarker* or "serum marker*" or algorithm* or score* or index* or model* or "risk score" or "diagnostic accuracy").ti,ab.	7369168

17	13 or 14 or 15 or 16	8762067

18	("NAFLD fibrosis score" or NFS or "FIB-4" or "FIB4" or "BARD score" or "ELF test" or "enhanced liver fibrosis" or FibroTest or FibroSure or FibroMeter or Hepascore or "MACK-3" or "ADAPT" or "Pro-C3" or "NIS4" or "Agile 3" or "Agile 4" or "FAST score" or MEFIB).ti,ab.	92967

19	("transient elastography" or "vibration-controlled transient elastography" or VCTE or FibroScan or "acoustic radiation force impulse" or ARFI or "shear wave elastography" or SWE or "2D-SWE" or "point-SWE" or "ultrasound elastography" or "magnetic resonance elastography" or MRE or MRI or "cT1" or "T1 mapping" or "LiverMultiScan").ti,ab.	384760

20	("artificial intelligence" or "machine learning" or radiomics).ti,ab.	217768

21	exp Artificial Intelligence/ or exp Machine Learning/	257491

22	18 or 19 or 20 or 21	834546

23	"liver biopsy".mp.	23723

24	(biopsy or histology or histologic or "reference standard" or "gold standard").ti,ab.	746668

25	23 or 24	746899

26	4 and 8 and 12 and (17 or 22) and 25	1533

**EMBASE Via Ovid**

1	exp nonalcoholic fatty liver disease/	85379

2	exp steatohepatitis/	20749

3	(NAFLD or NASH or MASLD or MAFLD or "metabolic dysfunction-associated steatotic liver disease").ti,ab.	77429

4	1 or 2 or 3	107641

5	exp liver fibrosis/	75980

6	exp hepatic cirrhosis/	217980

7	(fibrosis or fibrotic or "liver fibrosis" or "hepatic fibrosis" or cirrhosis or "liver cirrhosis" or F2 or F3 or F4).ti,ab.	673464

8	5 or 6 or 7	746213

9	exp obesity/	797833

10	exp body mass/	793983

11	exp body mass index/	793983

12	(obes* or BMI or "body mass index" or "morbid obes*" or "severe obes*" or "class I obes*" or "class II obes*" or "class III obes*" or bariatric or "weight loss surgery" or "bariatric surgery").ti,ab.	1157189

13	9 or 10 or 11 or 12	1587452

14	exp diagnostic accuracy/	357532

15	exp noninvasive diagnosis/	3788

16	exp biomarker/	622145

17	exp diagnostic test accuracy study/	322154

18	(noninvasive or "non-invasive" or biomarker* or "serum marker*" or algorithm* or score* or index* or model* or "risk score" or "diagnostic accuracy").ti,ab.	9909419

19	14 or 15 or 16 or 17 or 18	10307098

20	("NAFLD fibrosis score" or NFS or "FIB-4" or "FIB4" or "BARD score" or "ELF test" or "enhanced liver fibrosis" or FibroTest or FibroSure or FibroMeter or Hepascore or "MACK-3" or "ADAPT" or "Pro-C3" or "NIS4" or "Agile 3" or "Agile 4" or "FAST score" or MEFIB).ti,ab.	121028

21	("transient elastography" or "vibration-controlled transient elastography" or VCTE or FibroScan or "acoustic radiation force impulse" or ARFI or "shear wave elastography" or SWE or "2D-SWE" or "point-SWE" or "ultrasound elastography" or "magnetic resonance elastography" or MRE or MRI or "cT1" or "T1 mapping" or "LiverMultiScan").ti,ab.	685002

22	("artificial intelligence" or "machine learning" or radiomics).ti,ab.	259077

23	exp artificial intelligence/ or exp machine learning/	732796

24	20 or 21 or 22 or 23	1523082

25	exp liver biopsy/	80650

26	(biopsy or histology or histologic or "reference standard" or "gold standard").ti,ab.	1216207

27	25 or 26	1250813

28	4 and 8 and 13 and (19 or 24) and 27	6187

**Pubmed**

("Non-alcoholic Fatty Liver Disease"[Mesh] OR "Non-alcoholic Steatohepatitis"[Mesh] OR NAFLD OR NASH OR "metabolic dysfunction-associated steatotic liver disease" OR MASLD OR MAFLD)

AND

("Liver Fibrosis"[Mesh] OR "Liver Cirrhosis"[Mesh] OR fibrosis OR fibrotic OR cirrhosis OR "hepatic fibrosis" OR "liver fibrosis")

AND

("Obesity"[Mesh] OR "Body Mass Index"[Mesh] OR obes* OR "body mass index" OR BMI OR bariatric OR "bariatric surgery" OR "weight loss surgery")

AND

("Liver Biopsy"[Mesh] OR biopsy OR histology OR "reference standard" OR "gold standard")

AND

("noninvasive" OR "non-invasive" OR biomarker* OR algorithm* OR score* OR index* OR model* OR "risk score" OR "diagnostic accuracy" OR "NAFLD fibrosis score" OR "FIB-4" OR "BARD score" OR "ELF test" OR "enhanced liver fibrosis" OR FibroTest OR FibroSure OR FibroMeter OR Hepascore OR "MACK-3" OR ADAPT OR "Pro-C3" OR NIS4 OR "FAST score" OR "Agile 3" OR "Agile 4" OR MEFIB OR "transient elastography" OR VCTE OR FibroScan OR "shear wave elastography" OR SWE OR ARFI OR "magnetic resonance elastography" OR MRE OR MRI OR "cT1" OR "T1 mapping" OR "LiverMultiScan" OR "artificial intelligence" OR "machine learning" OR radiomics)

**Cochrane Library**

#1	(NAFLD OR NASH OR MASLD OR MAFLD OR "metabolic dysfunction associated steatotic liver disease")	6029

#2	(fibrosis OR fibrotic OR cirrhosis OR "liver fibrosis" OR "hepatic fibrosis")	30936

#3	(obes* OR "body mass index" OR BMI OR morbid NEXT obes* OR severe NEXT obes* OR bariatric OR "bariatric surgery" OR "weight loss surgery")	138316

#4	("noninvasive" OR non NEXT invasive OR biomarker* OR serum NEXT marker* OR algorithm* OR score* OR index* OR model* OR risk NEXT score OR diagnostic NEXT accuracy OR

"NAFLD fibrosis score" OR NFS OR "FIB-4" OR FIB4 OR "BARD score" OR "ELF test" OR enhanced NEXT liver NEXT fibrosis OR FibroTest OR FibroSure OR FibroMeter OR HepaScore OR

"MACK-3" OR ADAPT OR Pro-C3 OR NIS4 OR FAST NEXT score OR Agile NEXT 3 OR Agile NEXT 4 OR MEFIB OR

transient NEXT elastography OR vibration NEXT controlled NEXT transient NEXT elastography OR VCTE OR FibroScan OR shear NEXT wave NEXT elastography OR SWE OR 2D NEXT SWE OR point NEXT SWE OR ARFI OR ultrasound NEXT elastography OR

magnetic NEXT resonance NEXT elastography OR MRE OR MRI OR cT1 OR T1 NEXT mapping OR LiverMultiScan OR

artificial NEXT intelligence OR machine NEXT learning OR radiomics)

#5	(biopsy OR histology OR histologic OR "liver biopsy" OR "reference standard" OR "gold standard")

#6	MeSH descriptor: [Fatty Liver] explode all trees

#7	MeSH descriptor: [Liver Cirrhosis] explode all trees

#8	MeSH descriptor: [Obesity] explode all trees

#9	MeSH descriptor: [Biopsy] explode all trees

#10	#1 OR #6

#11	#2 OR #7

#12	#3 OR #8

#13	#5 OR #9

#14	#10 AND #11 AND #12 AND #4 AND #13

**Web of Science**

TS=(NAFLD OR NASH OR MASLD OR MAFLD OR "metabolic dysfunction associated steatotic liver disease" OR "nonalcoholic fatty liver disease" OR "non alcoholic fatty liver disease" OR steatohepatitis)

AND

TS=(fibrosis OR fibrotic OR cirrhosis OR "liver fibrosis" OR "hepatic fibrosis")

AND

TS=(obes* OR "body mass index" OR BMI OR morbid NEAR/1 obes* OR severe NEAR/1 obes* OR bariatric OR "bariatric surgery" OR "weight loss surgery")

AND

TS=("noninvasive" OR "non-invasive" OR biomarker* OR "serum marker*" OR algorithm* OR score* OR index* OR model* OR "risk score" OR "diagnostic accuracy" OR "NAFLD fibrosis score" OR NFS OR "FIB-4" OR FIB4 OR "BARD score" OR "ELF test" OR "enhanced liver fibrosis" OR FibroTest OR FibroSure OR FibroMeter OR HepaScore OR "MACK-3" OR ADAPT OR "Pro-C3" OR NIS4 OR "FAST score" OR "Agile 3" OR "Agile 4" OR MEFIB OR"transient elastography" OR "vibration-controlled transient elastography" OR VCTE OR FibroScan OR "shear wave elastography" OR SWE OR ARFI OR "magnetic resonance elastography" OR MRE OR MRI OR cT1 OR "T1 mapping" OR LiverMultiScan OR "artificial intelligence" OR "machine learning" OR radiomics)

AND

TS=(biopsy OR histology OR histologic OR "liver biopsy" OR "reference standard" OR "gold standard")

## 2. **SUPPLEMENTARY DATA**

### **eFigure 1. PRISMA Flowchart**

### **eFigure 2. Quality assessment**

**eFigure 2. Risk of bias assessment of included studies using the QUADAS-2 tool.** Traffic-light plot summarising the methodological quality of the included studies across the four QUADAS-2 domains: patient selection (D1), index test (D2), reference standard (D3), and flow and timing (D4). Each circle represents the judgement for an individual study within a given domain. Green circles indicate low risk of bias, yellow circles indicate uncertainty, and red circles indicate high risk of bias. The overall risk of bias judgement for each study is shown in the final column.

### **eTable 1. Study-level diagnostic accuracy data for detection of significant fibrosis (≥F2)**

| **Study** | **Test** | **Cutoff** | **TP** | **FP** | **FN** | **TN** | **Sens** | **Spec** | **LR+** | **LR-** |
| --- | --- | --- | --- | --- | --- | --- | --- | --- | --- | --- |
| **Agarwal**  **2021** | VCTE | 7.95 | 45 | 33 | 16 | 73 | 0.74 | 0.69 | 2.37 | 0.38 |
| **Alcantara-Diaz 2023** | 2D-SWE | 4.61 | 68 | 70 | 33 | 56 | 0.67 | 0.44 | 1.21 | 0.74 |
| **Ali**  **2022** | VCTE | 12.8 | 14 | 43 | 5 | 105 | 0.74 | 0.71 | 2.54 | 0.37 |
| **Barsamian**  **2020** | VCTE | 8.1 | 15 | 20 | 5 | 76 | 0.75 | 0.79 | 3.60 | 0.32 |
| **Bauer**  **2024** | 5C1-2D-SWE | 8.5 | 9 | 6 | 35 | 53 | 0.20 | 0.90 | 2.01 | 0.89 |
| **Bauer**  **2024** | 5C1-pSWE | 6.1 | 13 | 6 | 32 | 44 | 0.29 | 0.88 | 2.41 | 0.81 |
| **Bauer**  **2024** | DAX-2D-pSWE | 5.5 | 11 | 6 | 31 | 51 | 0.26 | 0.89 | 2.49 | 0.82 |
| **Bauer**  **2024** | DAX-pSWE | 4.4 | 9 | 6 | 34 | 47 | 0.21 | 0.89 | 1.85 | 0.89 |
| **Bauer**  **2024** | ELF | 7.7 | 35 | 43 | 5 | 2 | 0.88 | 0.04 | 0.92 | 2.81 |
| **Bauer**  **2024** | VCTE | 12 | 13 | 4 | 34 | 37 | 0.28 | 0.90 | 2.84 | 0.80 |
| **Chen**  **2017** | MRE | 3.5 | 23 | 5 | 5 | 44 | 0.82 | 0.90 | 8.05 | 0.20 |
| **Chen**  **2017** | VCTE | 7.8 | 23 | 11 | 5 | 38 | 0.82 | 0.78 | 3.66 | 0.23 |
| **DaSilva**  **2021** | APRI | 0.27 | 14 | 22 | 7 | 65 | 0.67 | 0.75 | 2.64 | 0.45 |
| **DaSilva**  **2021** | ARFI | 1.32 | 12 | 26 | 4 | 37 | 0.75 | 0.59 | 1.82 | 0.43 |
| **DaSilva**  **2021** | BARD | 2 | 17 | 56 | 4 | 31 | 0.81 | 0.36 | 1.26 | 0.53 |
| **DaSilva**  **2021** | FIB-4 | 0.85 | 16 | 21 | 5 | 66 | 0.76 | 0.76 | 3.16 | 0.31 |
| **DaSilva**  **2021** | Forn | 3.44 | 19 | 36 | 2 | 51 | 0.90 | 0.59 | 2.19 | 0.16 |
| **DaSilva**  **2021** | NFS | 0.37 | 11 | 26 | 10 | 61 | 0.52 | 0.70 | 1.75 | 0.68 |
| **Dominik**  **2025** | Auto pSWE | 5.8 | 8 | 7 | 29 | 90 | 0.22 | 0.93 | 3.00 | 0.84 |
| **Dominik**  **2025** | VCTE | 13.3 | 7 | 6 | 24 | 77 | 0.23 | 0.93 | 3.12 | 0.83 |
| **Eilenberg**  **2021** | FAST | 0.301 | 19 | 46 | 7 | 61 | 0.73 | 0.57 | 1.70 | 0.47 |
| **Eilenberg**  **2021** | VCTE | 6.3 | 26 | 56 | 7 | 65 | 0.79 | 0.54 | 1.70 | 0.39 |
| **Garg**  **2018** | VCTE | 7.25 | 21 | 19 | 9 | 27 | 0.70 | 0.59 | 1.69 | 0.51 |
| **Giraudi**  **2018** | EGFR | 102.5 | 12 | 27 | 1 | 31 | 0.92 | 0.53 | 1.98 | 0.14 |
| **Giraudi**  **2018** | EGFR/IGF2 | 58 | 10 | 15 | 3 | 43 | 0.77 | 0.74 | 2.97 | 0.31 |
| **Giraudi**  **2018** | FIB-4 | 0.78 | 9 | 21 | 4 | 37 | 0.69 | 0.64 | 1.91 | 0.48 |
| **Giraudi**  **2018** | FibroMeter | 51 | 10 | 21 | 3 | 37 | 0.77 | 0.64 | 2.12 | 0.36 |
| **Giraudi**  **2018** | IGF2 | 1.9 | 11 | 15 | 2 | 43 | 0.85 | 0.74 | 3.27 | 0.21 |
| **Giraudi**  **2022** | FCN-2 | 3650 | 45 | 34 | 12 | 144 | 0.79 | 0.81 | 4.13 | 0.26 |
| **Huang**  **2023** | APRI | 0.43 | 23 | 82 | 11 | 257 | 0.68 | 0.76 | 2.80 | 0.43 |
| **Huang**  **2023** | BARD | 0.24 | 10 | 18 | 24 | 321 | 0.29 | 0.95 | 5.54 | 0.75 |
| **Huang**  **2023** | FIB-4 | 0.46 | 23 | 75 | 11 | 264 | 0.68 | 0.78 | 3.06 | 0.42 |
| **Huang**  **2023** | HFS | 0.43 | 20 | 52 | 14 | 287 | 0.59 | 0.85 | 3.83 | 0.49 |
| **Huang**  **2023** | NFS | 0.38 | 19 | 60 | 15 | 279 | 0.56 | 0.82 | 3.16 | 0.54 |
| **Jamialahmadi 2019** | 2D-SWE | 6.6 | 9 | 23 | 3 | 55 | 0.75 | 0.71 | 2.54 | 0.35 |
| **Lassailly**  **2011** | FibroTest | 0.48 | 1 | 1 | 19 | 267 | 0.05 | 1.00 | 13.40 | 0.95 |
| **Lefere**  **2017** | VCAM-1 | 13.2 | 16 | 7 | 4 | 34 | 0.80 | 0.83 | 4.69 | 0.24 |
| **Nassif**  **2017** | BARD | ≥2 | 21 | 161 | 1 | 75 | 0.95 | 0.32 | 1.40 | 0.14 |
| **Naveau**  **2014** | VCTE | 7.6 | 16 | 17 | 6 | 61 | 0.73 | 0.78 | 3.34 | 0.35 |
| **Ooi**  **2017** | APRI | 0.2 | 19 | 47 | 4 | 31 | 0.83 | 0.40 | 1.37 | 0.44 |
| **Ooi**  **2017** | BARD | 2 | 19 | 52 | 4 | 26 | 0.83 | 0.33 | 1.24 | 0.52 |
| **Ooi**  **2017** | FIB-4 | 0.74 | 18 | 41 | 5 | 37 | 0.78 | 0.47 | 1.49 | 0.46 |
| **Ooi**  **2017** | Forn | 3.5 | 19 | 24 | 4 | 54 | 0.83 | 0.69 | 2.68 | 0.25 |
| **Ooi**  **2017** | NFS | -1.292 | 18 | 49 | 5 | 29 | 0.78 | 0.37 | 1.25 | 0.58 |
| **Soresi**  **2020** | BARD | ≥2 | 10 | 17 | 11 | 19 | 0.48 | 0.53 | 1.01 | 0.99 |
| **Soresi**  **2020** | FIB-4 | 0.41 | 14 | 12 | 7 | 24 | 0.67 | 0.67 | 2.00 | 0.50 |
| **Soresi**  **2020** | NFS | -1.455 | 6 | 4 | 15 | 32 | 0.29 | 0.89 | 2.57 | 0.80 |
| **Sun**  **2025** | APRI | 0.19 | 25 | 89 | 10 | 117 | 0.71 | 0.57 | 1.65 | 0.50 |
| **Sun**  **2025** | Auto pSWE | 1.02 | 26 | 72 | 9 | 134 | 0.74 | 0.65 | 2.13 | 0.40 |
| **Sun**  **2025** | FIB-4 | 0.69 | 15 | 23 | 20 | 183 | 0.43 | 0.89 | 3.84 | 0.64 |
| **Younossi**  **2023** | ELF | 9.8 | 80 | 24 | 100 | 259 | 0.44 | 0.92 | 5.24 | 0.61 |
| **Younossi**  **2023** | FIB-4 | 1.45 | 81 | 20 | 90 | 233 | 0.47 | 0.92 | 5.99 | 0.57 |

Abbreviations: TP = true positives; FP= false positives; FN= false negatives; TN =true negatives; Sens = sensitivity; Spec = specificity; LR+ = positive likelihood ratio; LR- = negative likelihood ratio. Values correspond to study-level 2×2 contingency tables used for the diagnostic test accuracy meta-analysis.

### **eTable 2. Study-level diagnostic accuracy data for detection of advanced fibrosis (≥F3)**

| **Study** | **Test** | **Cutoff** | **TP** | **FP** | **FN** | **TN** | **Sens** | **Spec** | **LR+** | **LR−** |
| --- | --- | --- | --- | --- | --- | --- | --- | --- | --- | --- |
| **Agarwal**  **2021** | VCTE | 8.95 | 23 | 37 | 7 | 100 | 0.77 | 0.73 | 2.84 | 0.32 |
| **Alcantara-Diaz 2023** | 2D-SWE | 5.14 | 16 | 88 | 2 | 121 | 0.89 | 0.58 | 2.11 | 0.19 |
| **Barsamian**  **2020** | VCTE | 8.7 | 10 | 20 | 1 | 85 | 0.91 | 0.81 | 4.77 | 0.11 |
| **Bauer**  **2024** | 5C1-2D-SWE | 9 | 5 | 10 | 3 | 85 | 0.62 | 0.89 | 5.94 | 0.42 |
| **Bauer**  **2024** | 5C1-pSWE | 8.5 | 6 | 8 | 2 | 79 | 0.75 | 0.91 | 8.16 | 0.28 |
| **Bauer**  **2024** | DAX-2D-pSWE | 9 | 6 | 4 | 1 | 88 | 0.86 | 0.96 | 19.71 | 0.15 |
| **Bauer**  **2024** | DAX-pSWE | 8.9 | 6 | 2 | 0 | 88 | 1.00 | 0.98 | 45.00 | 0.00 |
| **Bauer**  **2024** | ELF | 9.8 | 5 | 4 | 1 | 53 | 0.83 | 0.93 | 11.88 | 0.18 |
| **Bauer**  **2024** | VCTE | 13 | 9 | 10 | 1 | 96 | 0.90 | 0.91 | 9.54 | 0.11 |
| **Chen**  **2017** | MRE | 3.6 | 16 | 10 | 3 | 48 | 0.84 | 0.83 | 4.88 | 0.19 |
| **Chen**  **2017** | VCTE | 7.6 | 16 | 21 | 3 | 37 | 0.84 | 0.64 | 2.33 | 0.25 |
| **DeCarli**  **2020** | AAR | >1 | 4 | 52 | 24 | 203 | 0.14 | 0.80 | 0.70 | 1.08 |
| **DeCarli**  **2020** | APRI | 0.98 | 6 | 1 | 18 | 256 | 0.25 | 1.00 | 64.25 | 0.75 |
| **DeCarli**  **2020** | BAAT | >1 | 19 | 117 | 7 | 127 | 0.73 | 0.52 | 1.52 | 0.52 |
| **DeCarli**  **2020** | BARD | ≥2 | 16 | 144 | 12 | 111 | 0.57 | 0.44 | 1.01 | 0.98 |
| **DeCarli**  **2020** | FIB-4 | 2.67 | 8 | 7 | 18 | 233 | 0.31 | 0.97 | 10.55 | 0.71 |
| **DeCarli**  **2020** | NFS | 0.676 | 3 | 7 | 21 | 215 | 0.12 | 0.97 | 3.96 | 0.90 |
| **Dominik**  **2025** | Auto pSWE | 6.2 | 7 | 5 | 2 | 120 | 0.78 | 0.96 | 19.44 | 0.23 |
| **Dominik**  **2025** | VCTE | 15 | 6 | 6 | 2 | 100 | 0.75 | 0.94 | 13.25 | 0.27 |
| **Eilenberg**  **2021** | FAST | 0.714 | 4 | 8 | 5 | 116 | 0.44 | 0.94 | 6.89 | 0.59 |
| **Eilenberg**  **2021** | VCTE | 12.6 | 7 | 12 | 6 | 129 | 0.54 | 0.91 | 6.33 | 0.50 |
| **Garg**  **2018** | VCTE | 12.5 | 7 | 8 | 4 | 57 | 0.64 | 0.88 | 5.17 | 0.41 |
| **Jamialahmadi 2019** | 2D-SWE | 6.75 | 4 | 25 | 1 | 60 | 0.80 | 0.71 | 2.72 | 0.28 |
| **Marques**  **2021** | APRI | 0.31 | 6 | 28 | 4 | 69 | 0.60 | 0.71 | 2.08 | 0.56 |
| **Marques**  **2021** | FIB-4 | 0.75 | 9 | 51 | 1 | 46 | 0.90 | 0.47 | 1.71 | 0.21 |
| **Marques**  **2021** | IGF-1 | 98.83 | 7 | 37 | 3 | 60 | 0.70 | 0.62 | 1.84 | 0.49 |
| **Marques**  **2021** | NFS | -0.96 | 3 | 8 | 4 | 56 | 0.43 | 0.88 | 3.43 | 0.65 |
| **Naveau**  **2014** | VCTE | 7.6 | 9 | 24 | 0 | 67 | 1.00 | 0.74 | 3.79 | 0.00 |
| **Ooi**  **2017** | APRI | 0.405 | 3 | 8 | 0 | 90 | 1.00 | 0.92 | 12.25 | 0.00 |
| **Ooi**  **2017** | FIB-4 | 0.89 | 3 | 37 | 0 | 61 | 1.00 | 0.62 | 2.65 | 0.00 |
| **Ooi**  **2017** | Forn | 4.4 | 3 | 29 | 0 | 69 | 1.00 | 0.70 | 3.38 | 0.00 |
| **Ooi**  **2017** | NFS | 0.206 | 2 | 20 | 1 | 78 | 0.67 | 0.80 | 3.27 | 0.42 |
| **Pimentel**  **2010** | NFS | 0.676 | 20 | 3 | 2 | 133 | 0.91 | 0.98 | 41.21 | 0.09 |
| **Schmitz**  **2020** | APRI | 0.1 | 10 | 31 | 2 | 98 | 0.83 | 0.76 | 3.47 | 0.22 |
| **Schmitz**  **2020** | FIB-4 | 0.78 | 6 | 45 | 6 | 84 | 0.50 | 0.65 | 1.43 | 0.77 |
| **Schmitz**  **2020** | LiMAx | 292 | 7 | 54 | 5 | 75 | 0.58 | 0.58 | 1.39 | 0.72 |
| **Schmitz**  **2020** | NCS | 6 | 9 | 12 | 3 | 117 | 0.75 | 0.91 | 8.06 | 0.28 |
| **Schmitz**  **2020** | NFS | -0.44 | 7 | 49 | 5 | 80 | 0.58 | 0.62 | 1.54 | 0.67 |
| **Smiderle**  **2021** | NFS | 0.676 | 7 | 53 | 25 | 259 | 0.22 | 0.83 | 1.29 | 0.94 |
| **Smiderle**  **2021** | TyG | 8.91 | 21 | 131 | 13 | 218 | 0.62 | 0.62 | 1.65 | 0.61 |
| **Sun**  **2025** | APRI | 0.55 | 3 | 21 | 3 | 214 | 0.50 | 0.91 | 5.60 | 0.55 |
| **Sun**  **2025** | Auto pSWE | 1.3 | 5 | 2 | 1 | 233 | 0.83 | 0.99 | 97.92 | 0.17 |
| **Sun**  **2025** | FIB-4 | 0.8 | 4 | 47 | 2 | 188 | 0.67 | 0.80 | 3.33 | 0.42 |

Abbreviations: TP = true positives; FP= false positives; FN= false negatives; TN =true negatives; Sens = sensitivity; Spec = specificity; LR+ = positive likelihood ratio; LR- = negative likelihood ratio. Values correspond to study-level 2×2 contingency tables used for the diagnostic test accuracy meta-analysis.

### **Model diagnostics and influence analysis**

For significant fibrosis (≥F2), pooled estimates were numerically stable across the feasible alternative quadrature specifications. VCTE and NFS estimates were unchanged using 5, 7 and 9 quadrature points. FIB-4 estimates were unchanged using 5 and 7 points, whereas the 9-point fit failed because of an unstable Hessian. For APRI, the primary, 5-point and 9-point fits produced essentially identical estimates, whereas the 7-point fit failed because of an unstable Hessian. The estimated random-effects correlation was −1.00 in all four models, indicating boundary estimation of the variance–covariance structure. No study exceeded the post hoc Cook’s-distance heuristic of 20/*k*, and no absolute standardised empirical Bayes random effect exceeded 2.

Leave-one-study-out analyses were estimable for VCTE and FIB-4. For VCTE, pooled sensitivity ranged from 0.62 to 0.69, specificity from 0.73 to 0.79 and DOR from 5.36 to 6.33. For FIB-4, sensitivity ranged from 0.62 to 0.68, specificity from 0.73 to 0.80 and DOR from 5.49 to 6.72. No individual omission materially altered the pooled interpretation. Leave-one-study-out analyses were not estimable for NFS or APRI because omission of one study left fewer than four studies available for bivariate modelling. Q-Q plots showed mild tail deviations for VCTE and FIB-4 and no obvious gross departure from the assumed distribution for NFS or APRI; interpretation was limited by the small number of contributing studies.

For advanced fibrosis (≥F3), pooled estimates were likewise stable across feasible quadrature specifications. VCTE and NFS estimates were unchanged using 5, 7 and 9 quadrature points. For FIB-4, the standard 9-point fit failed because of an unstable Hessian, whereas the corresponding 9-point *gllamm* fit converged with essentially unchanged estimates. For APRI, the primary, 5-point and 9-point fits produced essentially identical estimates, whereas the 7-point fit failed because of an unstable Hessian. Random-effects correlation estimates were −1.00 for VCTE, FIB-4 and APRI and 0.084 for NFS.

Pimentel 2010 was the only ≥F3 study to exceed the Cook’s-distance heuristic, with a Cook’s distance of 3.83 against a threshold of 3.33 in the NFS model; however, its maximum absolute standardised empirical Bayes random effect was 1.70, and no study in any ≥F3 model exceeded the residual screening threshold of 2. Leave-one-study-out sensitivity, specificity and DOR ranges were 0.75-0.81, 0.82-0.86 and 15.51-22.67 for VCTE; 0.53-0.74, 0.65-0.82 and 4.91-10.47 for FIB-4; 0.30-0.56, 0.85-0.91 and 2.35-10.80 for NFS; and 0.50-0.71, 0.84-0.94 and 12.93-24.23 for APRI. Omission of Pimentel 2010 produced the largest change in the NFS model, reducing sensitivity and DOR, but did not alter the overall interpretation of relatively low sensitivity. Q-Q plots showed no gross departure from the assumed random-effects distribution, although tail deviations were apparent in some models and graphical assessment was limited by the small number of studies

### **eTable 3. Model diagnostics and influence analyses for detection of significant fibrosis (≥F2)**

| **Test** | **Studies** | **Sens/Spec** | **Numerical stability** | **Random-effects correlation** | **Maximum Cook's distance** | **Maximum absolute standardised residual** | **Leave-one-study-out** | **Q-Q plot** |
| --- | --- | --- | --- | --- | --- | --- | --- | --- |
| **VCTE** | 9 | 0.64 / 0.77 | Estimates unchanged using 5, 7 and 9 quadrature points. | −1.00 | 1.33 (Eilenberg 2021); threshold 2.22 | 1.93 (Dominik 2025) | Sensitivity 0.62-0.69; specificity 0.73-0.79; DOR 5.36-6.33. No individual omission materially altered the pooled result. | Mild tail deviation, without gross departure from linearity; assessment limited by nine studies. |
| **FIB-4** | 7 | 0.65 / 0.77 | Estimates unchanged using 5 and 7 quadrature points; the 9-point fit failed because of an unstable Hessian. | −1.00 | 0.81 (Younossi 2023); threshold 2.86 | 1.55 (Ooi 2017) | Sensitivity 0.62-0.68; specificity 0.73-0.80; DOR 5.49-6.72. No individual omission materially altered the pooled result. | Mild tail deviation, without gross departure from linearity; assessment limited by seven studies. |
| **NFS** | 4 | 0.56 / 0.74 | Estimates unchanged using 5, 7 and 9 quadrature points. | −1.00 | 1.27 (Huang 2023); threshold 5.00 | 1.52 (Ooi 2017) | Not estimable because omission of one study left fewer than four studies. | No obvious gross departure from linearity; assessment severely limited by four studies. |
| **APRI** | 4 | 0.72 / 0.63 | Primary, 5-point and 9-point quadrature fits produced essentially identical estimates; the 7-point fit failed because of an unstable Hessian. | −1.00 | 1.25 (Ooi 2017); threshold 5.00 | 1.48 (Ooi 2017) | Not estimable because omission of one study left fewer than four studies. | No obvious gross departure from linearity; assessment severely limited by four studies. |

**Notes.** Cook’s distance was assessed using a post-hoc heuristic threshold of 20/k, where k represents the number of contributing studies. Standardised empirical Bayes random effects with absolute values greater than 2 were considered potential residual outliers. Leave-one-study-out analyses were not estimable for models containing only four studies because *metandi* requires at least four studies. Q-Q plots were assessed graphically for gross departures from the assumed normal distribution; no formal normality tests were performed because of the small number of studies. Boundary estimates of the random-effects correlation should be interpreted cautiously and likely reflect limited identification of the variance–covariance structure in models with few studies and heterogeneous diagnostic thresholds.

### **eTable 4. Model diagnostics and influence analyses for detection of advanced fibrosis (≥F3)**

| **Test** | **Studies** | **Sens/Spec** | **Numerical stability** | **Random-effects correlation** | **Maximum Cook's distance** | **Maximum absolute standardised residual** | **Leave-one-study-out** | **Q-Q plot** |
| --- | --- | --- | --- | --- | --- | --- | --- | --- |
| **VCTE** | 8 | 0.77 / 0.84 | Estimates unchanged using 5, 7 and 9 quadrature points. | −1.00 | 1.24 (Eilenberg 2021); threshold 2.50 | 1.47 (Chen 2017) | Sensitivity 0.75-0.81; specificity 0.82-0.86; DOR 15.51-22.67. No individual omission materially altered the pooled result. | Mild deviation from normal distribution, without gross departure; assessment limited by eight studies. |
| **FIB-4** | 5 | 0.63 / 0.76 | Estimates unchanged using 5 and 7 quadrature points. The standard 9-point fit failed because of an unstable Hessian, whereas the 9-point gllamm fit converged with essentially unchanged estimates. | −1.00 | 2.08 (De Carli 2020); threshold 4.00 | 1.86 (De Carli 2020) | Sensitivity 0.53-0.74; specificity 0.65-0.82; DOR 4.91-10.47. Omission of Schmitz 2020 increased the DOR, indicating some influence on effect magnitude, but did not change the direction of the finding. | Tail deviation driven by De Carli 2020; assessment severely limited by five studies. |
| **NFS** | 6 | 0.47 / 0.89 | Estimates unchanged using 5, 7 and 9 quadrature points. | 0.084 | 3.83 (Pimentel 2010); threshold 3.33 | 1.70 (Pimentel 2010) | Sensitivity 0.30-0.56; specificity 0.85-0.91; DOR 2.35-10.80. Pimentel 2010 was influential; its omission reduced sensitivity to 0.30 and DOR to 2.35. | Mild tail deviation, with Pimentel 2010 contributing most prominently; no gross departure normal distribution, although assessment was limited by six studies. |
| **APRI** | 5 | 0.59 / 0.92 | Primary, 5-point and 9-point quadrature fits produced essentially identical estimates; the 7-point fit failed because of an unstable Hessian. | −1.00 | 2.20 (De Carli 2020); threshold 4.00 | 1.91 (De Carli 2020) | Sensitivity 0.50-0.70; specificity 0.84-0.94; DOR 12.93-24.23. No individual omission materially altered the pooled result. | Tail deviation driven by De Carli 2020; assessment severely limited by five studies. |

**Notes**. Cook’s distance was assessed using a post-hoc heuristic threshold of 20/k, where k represents the number of contributing studies. Standardised empirical Bayes random effects with absolute values greater than 2 were considered potential residual outliers. Leave-one-study-out analyses were performed when omission left at least four studies. Q-Q plots were assessed graphically for gross departures from linearity; no formal normality tests were performed because of the small number of studies. Boundary estimates of the random-effects correlation should be interpreted cautiously and likely reflect limited identification of the variance–covariance structure in models with few studies and heterogeneous diagnostic thresholds.

### **eTable 5. Technical feasibility, non-evaluable examinations and indeterminate classifications of NITs**

**Panel A. Technical feasibility of imaging-based fibrosis tests**

| **Study** | **Modality / probe** | **Attempted, n** | **Technical failure** | **Unreliable / invalid** | **Overall non-evaluable** | **Definition and principal feasibility finding** |
| --- | --- | --- | --- | --- | --- | --- |
| **Agarwal 2021** | VCTE; XL | 179 | 12/179 (6.7%) | NR | 12/179 (6.7%) | Valid TE not obtained. Failure was associated with higher BMI and abdominal girth. |
| **Ali 2022** | VCTE; XL | 177 | 5/177 (2.8%) incomplete | 79/167 (47.3%) | Not calculable | Reliability required ≥10 valid measurements, success rate ≥60%, and IQR/median <30%. 88/167 (52.7%) analysed complete-data examinations met all criteria. |
| **Barsamian 2020** | VCTE; M/XL | 119 | 2/119 (1.7%) | 7/117 (6.0%) | 9/119 (7.6%) | Failure: <10 valid measurements. Unreliable: IQR/median >0.30 when LSM ≥7.1 kPa. BMI was higher in unreliable examinations. |
| **Bauer 2024** | VCTE; M/XL | 129 | 13/129 (10.1%) | 14/116 (12.1%) | 27/129 (20.9%) | Failure: required measurements not obtained within ≥30 attempts. Unreliable: IQR/median >30% when LSM ≥7.1 kPa. |
| **Bauer 2024** | pSWE; 5C1 | 129 | 0/129 (0%) | 34/129 (26.4%) | 34/129 (26.4%) | No technical failures; reliability required IQR/median ≤30%. |
| **Bauer 2024** | 2D-SWE; 5C1 | 129 | 0/129 (0%) | 26/129 (20.2%) | 26/129 (20.2%) | No technical failures; reliability required IQR/median ≤30%. |
| **Bauer 2024** | pSWE; DAX | 129 | 0/129 (0%) | 33/129 (25.6%) | 33/129 (25.6%) | No technical failures; reliability required IQR/median ≤30%. DAX eliminated acquisition failure but not unreliable results. |
| **Bauer 2024** | 2D-SWE; DAX | 129 | 0/129 (0%) | 30/129 (23.3%) | 30/129 (23.3%) | No technical failures; reliability required IQR/median ≤30%. DAX eliminated acquisition failure but not unreliable results. |
| **Chen 2017** | MR elastography | 110 | 5/110 (4.5%) | NR | 5/110 (4.5%) | Two patients could not fit in the 60-cm bore and three developed claustrophobia; unsuccessful examinations had greater waist circumference. |
| **Chen 2017** | VCTE; M/XL | 97 | 4/97 (4.1%) | 14/97 (14.4%) | 18/97 (18.6%) | Failure: <10 valid measurements. Strict reliability required success rate ≥60% and IQR/median ≤30%. Higher BMI, chest and waist circumference were associated with non-evaluable examinations. |
| **Dominik 2025** | Auto-pSWE; DAX | 134 | 0/134 (0%) | 0/134 (0%) | 0/134 (0%) | All examinations were reported successful and reliable. |
| **Dominik 2025** | VCTE; M/XL | 134 | 7/134 (5.2%) | 2/134 (1.5%) | 9/134 (6.7%) | Two unreliable examinations were inferred from the reported patient flow. |
| **Eilenberg 2021** | VCTE; XL | 170 | Not separately reported | 16/170 (9.4%) | 16/170 (9.4%) | Unreliable VCTE was defined as IQR/median >30% for LSM >7.0 kPa. Unreliable results were more frequent at higher BMI. |
| **Garg 2018** | VCTE; XL | 124 | 15/124 (12.1%) | NR | 15/124 (12.1%) | Failure: inability to obtain valid LSM/CAP. Failure was associated with higher BMI and abdominal girth. |
| **Jamialahmadi 2019** | 2D-SWE; SC6-1 | 111 | 3/111 (2.7%) | NR | 3/111 (2.7%) | All failures were attributed to excessive subcutaneous adipose tissue. |
| **Naveau 2014** | VCTE; M/XL | 173 | 15/136 (11%) | 11/136 (8.1%) | 26/136 (19.1%) | 22 had <10 valid measurements, including 15 complete failures; 4/136 (2.9%) were unreliable. BMI was higher in failed/unreliable examinations. |
| **DaSilva 2021** | ARFI; convex probe | 108 | 20/108 (18.5%) | 9/108 (8.3%) | 29/108 (26.9%) | Failure: <6 valid measurements; unreliable: IQR/median >30%. Non-evaluable rates were higher at BMI ≥42 kg/m². |
| **Sun 2025** | Multisample Auto-pSWE; DAX | 253 | 4/253 (1.6%) | NR | 4/253 (1.6%) | Four examinations were unsuccessful because of poor respiratory cooperation or excessive subcutaneous fat. Satisfactory measurements required IQR/median ≤15% |

**Panel B. Availability and indeterminate classification of serum-based tests**

| **Study** | **Test** | **Available / calculable** | **Thresholds defining the intermediate range or decision rules** | **Indeterminate / unresolved** | **Source of estimate** | **Qualification** |
| --- | --- | --- | --- | --- | --- | --- |
| **Ooi 2017** | FIB-4 | 101/101 (100%) | 1.30–3.25 | 9/101 (8.9%) | Reconstructed | Derived from published low- and high-threshold classification counts. |
| **Ooi 2017** | Forn index | 101/101 (100%) | 4.2–6.9 | 34/101 (33.7%) | Reconstructed | Derived from published low- and high-threshold classification counts. |
| **Ooi 2017** | NFS | 101/101 (100%) | −1.455 to 0.676 | 60/101 (59.4%) | Reconstructed | Derived from published low- and high-threshold classification counts. |
| **Pimentel 2010** | NFS | 158/158 (100%) | −1.455 to 0.676 | 25/158 (15.8%) | Directly reported | 1/25 indeterminate patients had advanced fibrosis. |
| **Younossi 2023** | ELF | 463/463 complete cases | 9.8 to <11.3 | 18%; exact n NR | Directly reported percentage | Broader NAFLD cohort; mean BMI was 40.7 kg/m². |
| **Younossi 2023** | FIB-4 | 424/463 (91.6%) | 1.45 to <3.25 | 78/424 (18.4%) | Reconstructed | Broader NAFLD cohort; mean BMI was 40.7 kg/m². |
| **Younossi 2023** | ELF + FIB-4 combined rules | 424 paired complete cases | Rule out: ELF ≤7.7 or FIB-4 ≤0.30; rule in: ELF ≥9.8 and FIB-4 ≥1.96 | 326/424 (76.9%) | Reconstructed | Operationally unresolved, not labelled indeterminate; reconstructed from the reported rule-in and rule-out operating characteristics |
| **Bauer 2024** | FIB-4 | 123/129 (95.3%) | Single threshold 1.3 | Not applicable | Directly reported availability | Six participants lacked an available/calculable FIB-4 result. |
| **Barsamian 2020** | FIB-4, NFS and FibroMeter-NAFLD | 75/116 (64.7%) calculable together | No classification thresholds reported; analysed by AUROC | Not estimable | Directly reported joint availability | Individual score-specific missingness was not reported. |
| **De Carli 2020** | APRI / BARD / FIB-4 / NFS | 281/323 (87.0%) / 283/323 (87.6%) / 266/323 (82.4%) / 246/323 (76.2%) | Single thresholds | Not applicable | Directly reported denominators | Availability varied because required clinical or laboratory components were missing. |
| **Marques 2021** | APRI / FIB-4 / NFS | 107/203 (52.7%) / 107/203 (52.7%) / 71/203 (35.0%) | Single thresholds | Not applicable | Reported complete-case denominators | The reasons for missing score components and the reduced complete-case denominators were not reported. |
| **Smiderle 2021** | NFS | 344/423 (81.3%) | −1.455 to 0.676 | 112/344 (32.6%) | Reconstructed from published operating characteristics at both thresholds | Denominator was reconstructed. Reasons for unavailable NFS results were not reported. |

Studies describing dual-threshold serum-score ranges without sufficient cohort-level category data to estimate indeterminate proportions included Schmitz 2020 and Soresi 2020. Schmitz described standard FIB-4 and NFS dual thresholds but analysed fibrosis using study-specific single ROC-derived cut-offs; Soresi defined the NFS indeterminate range (−1.455 to 0.676) but did not report the numbers of patients in the low-, intermediate-, and high-risk categories. Therefore, indeterminate proportions could not be estimated for these studies.

### **eTable 6. Liver biopsy details**

Post hoc sensitivity analyses examined whether potential wedge or subcapsular fibrosis over-staging influenced pooled diagnostic accuracy. The principal restriction excluded studies with known unmitigated wedge or marginal-wedge sampling and studies in which wedge and core sampling were not separable, while retaining studies with explicit needle/core sampling, explicit mitigation of subcapsular fibrosis, or incompletely reported biopsy technique. The strict restriction retained studies with explicit needle/core sampling or explicit mitigation of subcapsular fibrosis; for ≥F2 VCTE, this was effectively a needle/core-only restriction.

For ≥F2 VCTE, the primary nine-study analysis yielded sensitivity 0.64 (95% CI 0.49-0.77), specificity 0.77 (0.66-0.85) and DOR 5.92 (4.17-8.40). After exclusion of known higher-risk sampling, seven studies remained, with sensitivity 0.60 (0.42-0.76), specificity 0.78 (0.65-0.87) and DOR 5.29 (3.61-7.75). Under the strict six-study restriction, sensitivity was 0.58 (0.37-0.76), specificity 0.77 (0.62-0.88) and DOR 4.78 (3.17-7.20). Thus, restriction of the biopsy reference standard produced a modest reduction in sensitivity, with little change in specificity or overall discrimination.

For ≥F3 VCTE, the primary eight-study estimates were sensitivity 0.77 (95% CI 0.67-0.85), specificity 0.84 (0.76-0.90) and DOR 18.12 (10.52-31.22). Exclusion of known higher-risk sampling left seven studies and yielded sensitivity 0.77 (0.62-0.87), specificity 0.86 (0.79-0.91) and DOR 20.31 (10.04-41.11). Under the strict six-study restriction, the corresponding estimates were 0.74 (0.62-0.84), 0.87 (0.79-0.92) and 18.74 (10.08-34.85). Overall VCTE discrimination therefore remained broadly stable across the biopsy-reference-standard restrictions.

For ≥F3 NFS, the primary six-study analysis yielded sensitivity 0.47 (95% CI 0.21-0.75), specificity 0.89 (0.76-0.95) and DOR 7.14 (1.47-34.79). Exclusion of known higher-risk sampling left five studies, with sensitivity 0.44 (0.16-0.77), specificity 0.92 (0.82-0.96) and DOR 8.80 (1.37-56.51). These estimates were highly imprecise. The strict ≥F3 NFS subset contained only three heterogeneous studies and was not pooled. Other restricted serum-score subsets, including APRI, FIB-4 and BARD, also contained too few studies for stable bivariate modelling and were not pooled. Overall, the analyses did not suggest that pooled VCTE performance was materially driven by wedge or subcapsular biopsy sampling; the NFS results were too imprecise to support a firm conclusion regarding biopsy method. Small-study VCTE models showed boundary or near-boundary random-effects correlation estimates, and these post hoc findings should therefore be interpreted as sensitivity analyses rather than definitive subgroup effects or evidence of equivalence between biopsy techniques.

**Panel A. Study-level biopsy classification**

| **Study** | **Biopsy technique** | **Subcapsular handling** | **Reference-standard classification** | **Principal analysis** | **Strict analysis** | **Key qualification** |
| --- | --- | --- | --- | --- | --- | --- |
| **Agarwal 2021** | 16G Tru-cut needle core | NR | Needle/core | Included | Included | Left-lobe intraoperative core; ≥1.5 cm and ≥6 portal tracts required. |
| **Alcantara-Diaz 2023** | Wedge biopsy | No mitigation reported | Unmitigated wedge | Excluded | Excluded | Biopsy mainly from the right hepatic border. |
| **Ali 2022** | Wedge biopsy | No mitigation reported | Unmitigated wedge | Excluded | Excluded | Standardised left-lobe wedge biopsy. |
| **Barsamian 2020** | Technique not specified | NR | Technique unclear | Included | Excluded | Intraoperative left-lobe biopsy; specimen quality assessed. |
| **Bauer 2024** | Needle core biopsy | NR | Needle/core | Included | Included | Direct-vision left-lobe core; typically 10–15 mm. |
| **Chen 2017** | Needle or subcapsular wedge | Subcapsular wedge used | Mixed | Excluded | Excluded | Biopsy-type-specific diagnostic results were not separable. |
| **DaSilva 2021** | Tru-cut needle biopsy | NR | Needle/core | Included | Included | Intraoperative biopsy. |
| **De Carli 2020** | Technique not specified | NR | Technique unclear | Included | Excluded | Intraoperative biopsy. |
| **Dominik 2025** | Surgical/percutaneous/  transjugular core biopsy | NR | Needle/core | Included | Included | No wedge biopsy described; median 8 portal fields. |
| **Eilenberg 2021** | 14G Tru-cut needle biopsy | NR | Needle/core | Included | Included | Left-lobe core; ≥1.5 cm and ≥6 portal fields. |
| **Garg 2018** | 16G Tru-cut needle core | NR | Needle/core | Included | Included | Intraoperative core; ≥1.5 cm and ≥6 portal tracts required. |
| **Giraudi 2018** | Wedge biopsy | No mitigation reported | Unmitigated wedge | Excluded | Excluded | Left-lobe wedge biopsy. |
| **Giraudi 2022** | Wedge biopsy | No mitigation reported | Unmitigated wedge | Excluded | Excluded | Surgical wedge biopsy. |
| **Huang 2023** | Wedge biopsy | No mitigation reported | Unmitigated wedge | Excluded | Excluded | Left-lobe wedge; specimen required ≥10 mm or ≥10 portal tracts. |
| **Jamialahmadi 2019** | 16G Tru-cut needle biopsy | NR | Needle/core | Included | Included | Left-lobe direct-vision core. |
| **Lassailly 2011** | Technique not specified | NR | Technique unclear | Included | Excluded | Operative biopsy; sampling instrument, lobe and depth not reported. |
| **Lefere 2017** | Technique not specified | NR | Technique unclear | Included | Excluded | Sampling instrument, lobe and depth not reported; ≥6 complete portal tracts required. |
| **Marques 2021** | Surgical biopsy; technique not specified | Fibrosis assessed >0.5 cm from capsule | Mitigated | Included | Included | Fibrosis assessed >0.5 cm from the liver capsule. |
| **Nassif 2017** | 16G suction needle biopsy during surgery | NR | Needle/core | Included | Included | Intraoperative needle biopsy. |
| **Naveau 2014** | 14G Tru-cut needle biopsy | NR | Needle/core | Included | Included | Left-lobe core; ≥10 mm or ≥10 portal tracts. |
| **Ooi 2017** | 14–16G Temno needle biopsy | NR | Needle/core | Included | Included | Mean core length 15.4 mm; mean 14.7 portal tracts. |
| **Pimentel 2010** | Wedge biopsy | Subcapsular fibrosis excluded from scoring | Mitigated | Included | Included | Subcapsular fibrosis explicitly excluded from scoring to avoid fibrosis overestimation. |
| **Schmitz 2020** | Wedge resection | No mitigation reported | Unmitigated wedge | Excluded | Excluded | Left-lobe wedge resection. |
| **Smiderle 2021** | Technique not specified | NR | Technique unclear | Included | Excluded | Intraoperative biopsy; sampling technique, lobe and depth not reported. |
| **Soresi 2020** | Wedge or core biopsy | Types not separable | Mixed | Excluded | Excluded | Wedge or core biopsy; sampling extended to the depth of Glisson's capsule and biopsy-type-specific results were not reported. |
| **Sun 2025** | Marginal wedge biopsy | No mitigation reported | Unmitigated wedge | Excluded | Excluded | Wedge taken from the left-lobe margin. |
| **Younossi 2023** | Technique not reported | NR | Technique unclear | Included | Excluded | Biopsy within 6 months of biomarker sampling; approach, instrument, lobe and adequacy not reported. |

**Panel B. Post hoc biopsy-method sensitivity analyses**

| **Endpoint / test** | **Analysis** | **Studies, n** | **Sensitivity (95% CI)** | **Specificity (95% CI)** | **DOR (95% CI)** | **Interpretation** |
| --- | --- | --- | --- | --- | --- | --- |
| **≥F2 VCTE** | Primary | 9 | 0.64 (0.49-0.77) | 0.77 (0.66–0.85) | 5.92 (4.17-8.40) | Reference |
| **≥F2 VCTE** | Exclude known high-risk sampling | 7 | 0.60 (0.42-0.76) | 0.78 (0.65-0.87) | 5.29 (3.61-7.75) | No material change |
| **≥F2 VCTE** | Strict low-risk reference standard | 6 | 0.58 (0.37-0.76) | 0.77 (0.62-0.88) | 4.78 (3.17-7.20) | Sensitivity slightly lower; specificity unchanged |
| **≥F3 VCTE** | Primary | 8 | 0.77 (0.67-0.85) | 0.84 (0.76-0.90) | 18.12 (10.52-31.22) | Reference |
| **≥F3 VCTE** | Exclude known high-risk sampling | 7 | 0.77 (0.62-0.87) | 0.86 (0.79-0.91) | 20.31 (10.04-41.11) | Sensitivity stable; specificity slightly higher |
| **≥F3 VCTE** | Strict low-risk reference standard | 6 | 0.74 (0.62-0.84) | 0.87 (0.79-0.92) | 18.74 (10.08-34.85) | Overall discrimination stable |
| **≥F3 NFS** | Primary | 6 | 0.47 (0.21-0.75) | 0.89 (0.76-0.95) | 7.14 (1.47-34.79) | Reference |
| **≥F3 NFS** | Exclude known high-risk sampling | 5 | 0.44 (0.16-0.77) | 0.92 (0.82-0.96) | 8.80 (1.37-56.51) | Specificity slightly higher; estimates very imprecise |

**Principal analysis**: excluded studies with known unmitigated wedge/marginal-wedge or mixed wedge/core sampling; retained explicit needle/core sampling, explicit subcapsular mitigation, and technique-unclear studies.

**Strict analysis:** retained explicit needle/core sampling or explicit mitigation of subcapsular fibrosis. For ≥F2 VCTE, this was effectively a needle/core-only restriction.

**Interpretation:** Exclusion of studies with known higher-risk biopsy sampling produced no material change in pooled VCTE diagnostic performance at either ≥F2 or ≥F3. Under the strict reference-standard restriction, VCTE sensitivity was modestly lower, while specificity was essentially unchanged or slightly higher and DORs remained comparable with the primary analyses. For ≥F3 NFS, exclusion of known higher-risk sampling was associated with slightly higher specificity, but sensitivity remained low and confidence intervals were wide. Overall, the analyses did not suggest that pooled VCTE performance was materially driven by wedge/subcapsular biopsy sampling; the NFS estimates were too imprecise for a firm conclusion regarding biopsy method.

The strict ≥F3 NFS subset contained only three heterogeneous studies and was not pooled. APRI, FIB-4 and BARD also had too few studies after restriction for stable bivariate modelling and were not pooled.

Small-study VCTE models showed boundary or near-boundary random-effects correlation estimates; these post hoc results should therefore be interpreted as sensitivity analyses rather than definitive subgroup effects or evidence of equivalence between biopsy techniques. The reference-standard classifications used for these post hoc sensitivity analyses were developed specifically to evaluate potential bias from wedge or subcapsular biopsy sampling and should not be interpreted as replacements for the formal QUADAS-2 reference-standard domain judgements.

### **eTable 7. Post-hoc VCTE threshold sensitivity analyses**

| **Endpoint / test** | **Analysis** | **Threshold range (kPa)** | **Studies, n** | **Sensitivity (95% CI)** | **Specificity (95% CI)** | **DOR (95% CI)** | **Interpretation / numerical stability** |
| --- | --- | --- | --- | --- | --- | --- | --- |
| **≥F2 VCTE** | Primary | 6.30-13.30 | 9 | 0.64 (0.49-0.77) | 0.77 (0.66-0.85) | 5.92 (4.17-8.40) | Reference |
| **≥F2 VCTE** | Lower-threshold cluster | 6.30-8.10 | 6 | 0.75 (0.69-0.81) | 0.70 (0.61-0.77) | 7.09 (4.23-11.88) | Higher sensitivity and lower specificity, consistent with the expected threshold trade-off; estimates essentially unchanged using 5, 7 and 9 quadrature points. Random-effects correlation at the +1 boundary. |
| **≥F3 VCTE** | Primary | 7.60-15.00 | 8 | 0.77 (0.67-0.85) | 0.84 (0.76-0.90) | 18.12 (10.52-31.22) | Reference |
| **≥F3 VCTE** | Lower-threshold cluster | 7.60-8.95 | 4 | 0.85 (0.72-0.92) | 0.74 (0.68-0.79) | 15.73 (6.53-37.89) | Higher sensitivity and lower specificity; estimates essentially unchanged using 5, 7 and 9 quadrature points. Random-effects correlation at the +1 boundary; exploratory because only four studies contributed. |
| **≥F3 VCTE** | Higher-threshold cluster | 12.50-15.00 | 4 | Not stably estimable | Not stably estimable | Not stably estimable | The standard bivariate fit failed during variance–covariance matrix inversion, and the alternative gllamm fit exceeded the iteration limit. |

**Notes.** Study-specific VCTE thresholds were summarised descriptively and were not treated as validated obesity-specific cut-offs. For ≥F2, the median threshold was 7.95 kPa (IQR 7.60-12.00; range 6.30-13.30). For ≥F3, the median threshold was 10.73 kPa (IQR 8.15-12.80; range 7.60-15.00). Threshold-restricted analyses were post hoc sensitivity analyses intended to assess the expected sensitivity–specificity trade-off rather than to identify an optimal threshold. The three ≥F2 studies using thresholds ≥12.0 kPa were not pooled separately because fewer than four studies were available. For ≥F3, the lower and higher threshold clusters each contained four studies; the higher-threshold subset could not be stably estimated. Boundary random-effects correlation estimates and the small number of studies warrant cautious interpretation.

**Abbreviations**: CI, confidence interval; DOR, diagnostic odds ratio; VCTE, vibration-controlled transient elastography.
